# Supplementary material for: Electrocatalytic hydrogenation of acetonitrile to ethylamine in acid
Source: Nat Commun. 2024 Apr 15;15:3233. doi: 10.1038/s41467-024-47622-9 (PMC11018601; doi:10.1038/s41467-024-47622-9)
Supplement: Supplementary file 1 — Supplementary Information [file 41467_2024_47622_MOESM1_ESM.pdf]

Supplementary Information

Electrocatalytic hydrogenation of acetonitrile to ethylamine in acid

Chongyang Tang[1,4], Cong Wei[2,4], Yanyan Fang[2,4], Bo Liu[2], Xianyin Song[1], Zenan Bian[2], Xuanwei Yin[2], Hongbo Wang[1], Zhaohui Liu[2], Gongming Wang[2]*, Xiangheng Xiao[1]* and Xiangfeng Duan[3]*

[1]School of Physics and Technology, Wuhan University, Wuhan, 430072, P. R. China.

[2]School of Chmistry and Materials Science, University of Science and Technology of China, Hefei, 230026, P. R. China.

[3]Department of Chemistry and Biochemistry, University of California, Los Angeles, Los Angeles, CA, USA.

[4]These authors contributed equally: Chongyang Tang, Cong Wei, Yanyan Fang

*Corresponding authors. Email: wanggm@ustc.edu.cn; xxh@whu.edu.cn; xduan@chem.ucla.edu

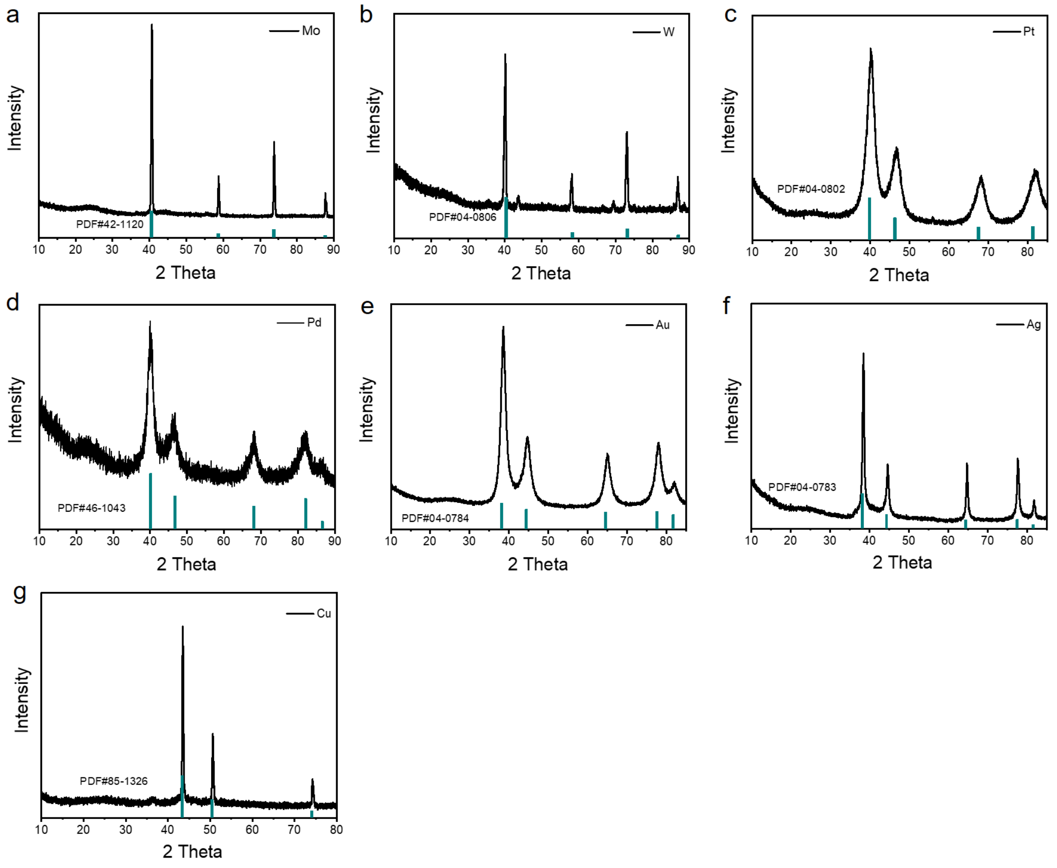

Supplementary Fig. 1. XRD characterization. PXRD patterns of Mo/C (**a**), W/C (**b**), Pt/C(**c**), Pd/C(**d**), Au/C(**e**), Ag/C (**f**) and Cu/C (**g**) catalysts.

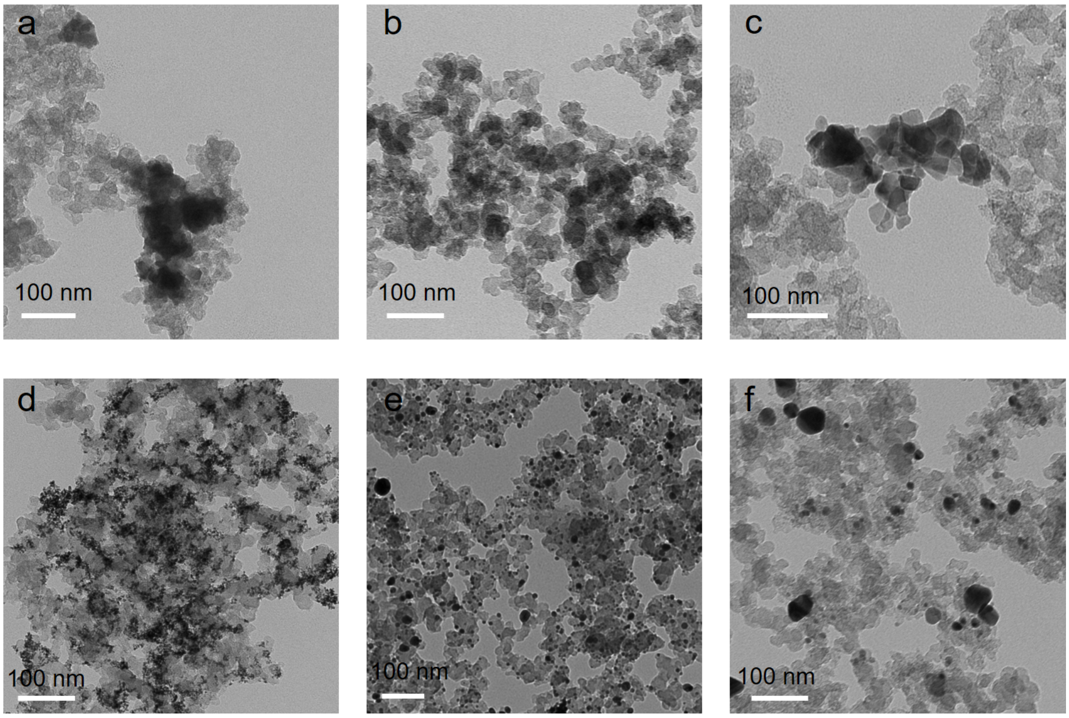

Supplementary Fig. 2. TEM characterization. TEM images of W (**a**), Mo (**b**), Cu (**c**), Pt (**d**), Au (**e**) and Ag (**f**) catalysts.

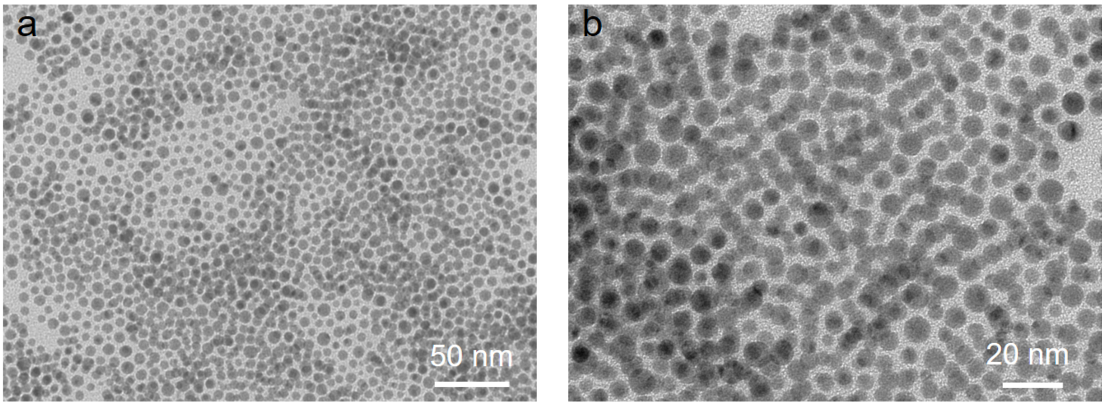

Supplementary Fig. 3. TEM characterization. a, b, TEM images of Pd nanoparticles.

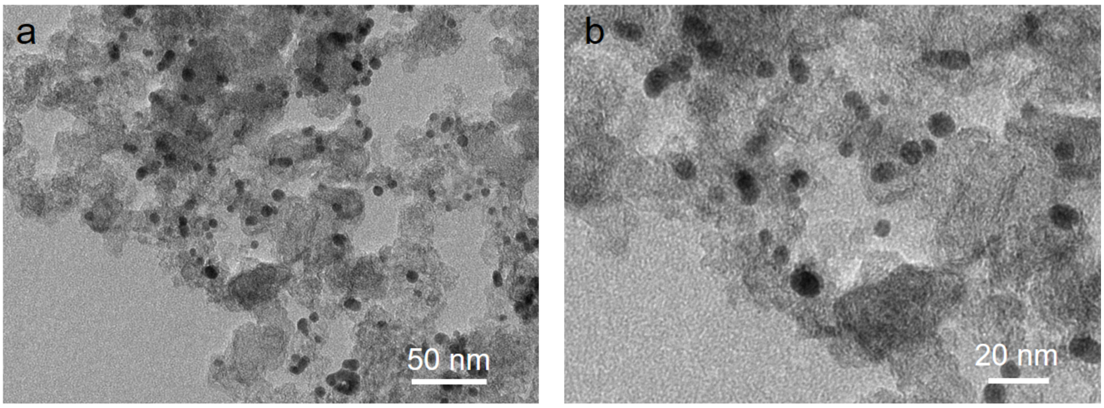

Supplementary Fig. 4. TEM characterization. a, b, TEM images of carbon supported Pd nanoparticles.

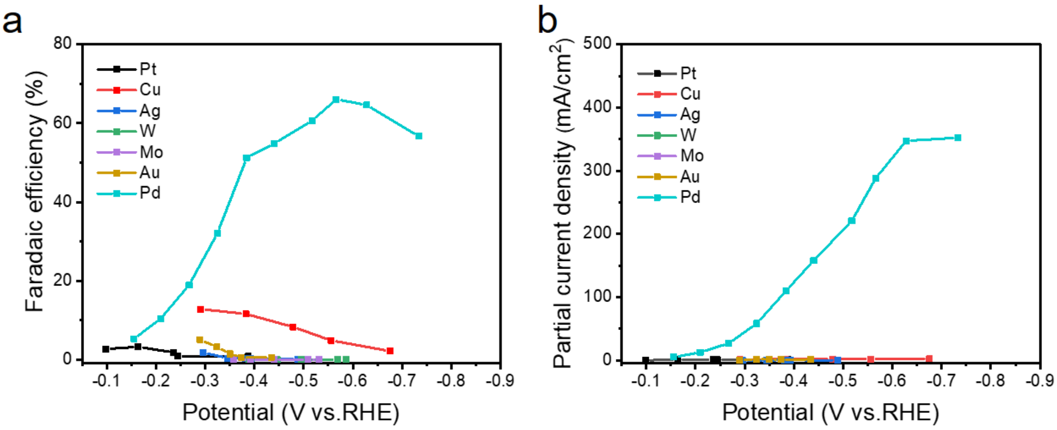

Supplementary Fig. 5. Activity of acetonitrile electroreduction over various catalysts. a, Potential-dependent ethylamine faradic efficiency and **b,** potential-dependent ethylamine partial current density on various metal catalysts.

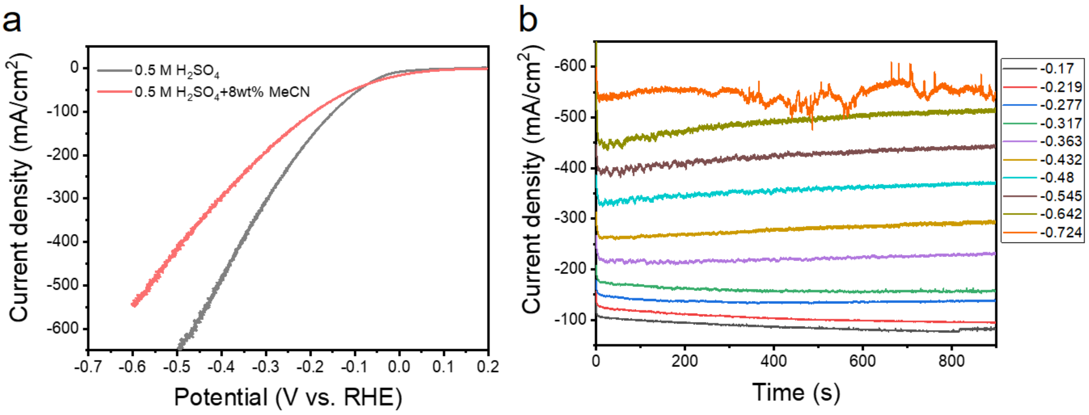

Supplementary Fig. 6. Electrochemical tests of Pd/C. a, The iR-corrected LSV curves of Pd/C obtained in 0.5 M H_2SO_4 and 0.5 M H_2SO_4 containing 8 wt% acetonitrile as electrolyte. **b,** The electrochemical reduction current of Pd/C at different potential for 900 s.

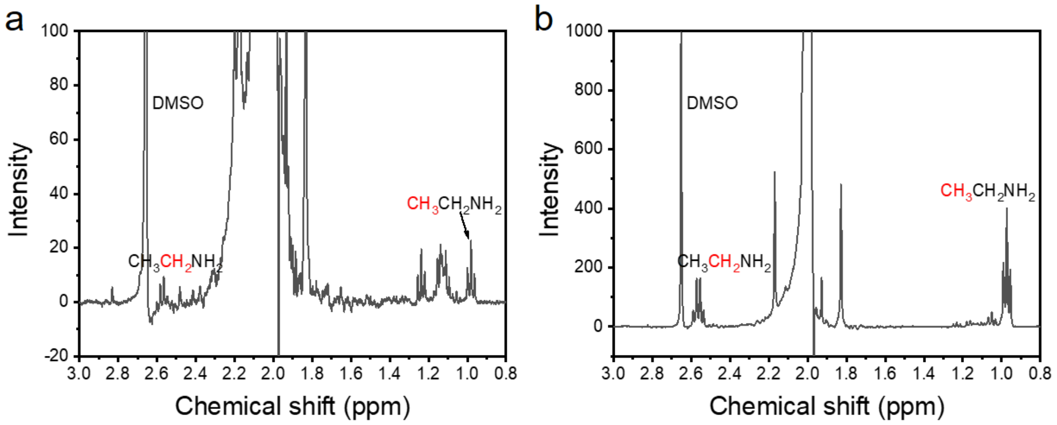

Supplementary Fig. 7. NMR spectra. The ^{1}H NMR spectra of electrolyte products by Pd/C at -0.267 V (**a**) and -0.734 V (**b**) for 900s.

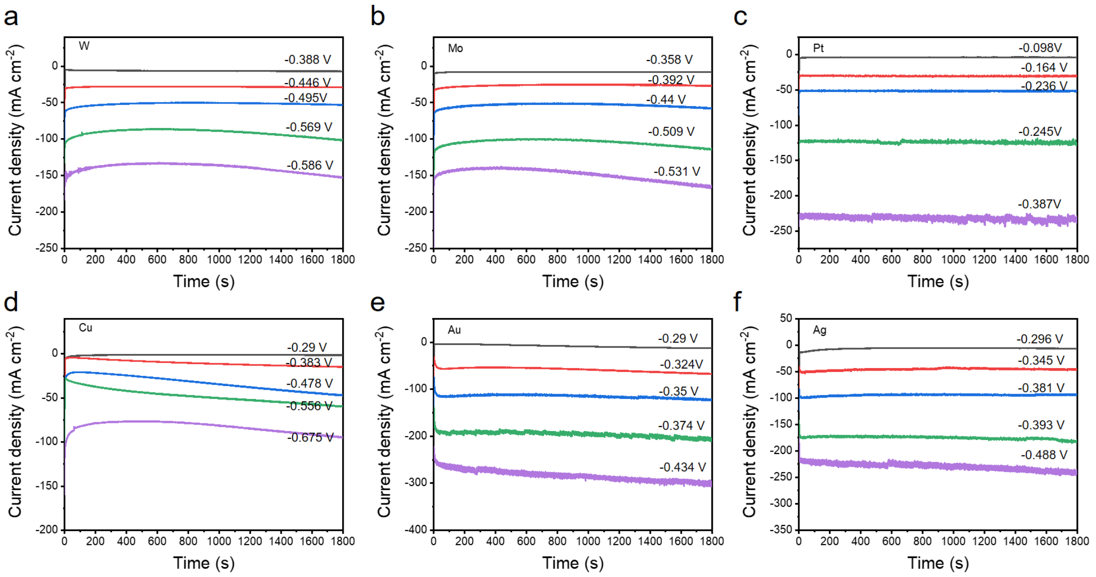

Supplementary Fig. 8. *J*-t curves of different catalysts. a, W/C, **b,** Mo/C, **c,** Pt/C, **d,** Cu/C, **e,** Au/C and **f,** Ag/C.

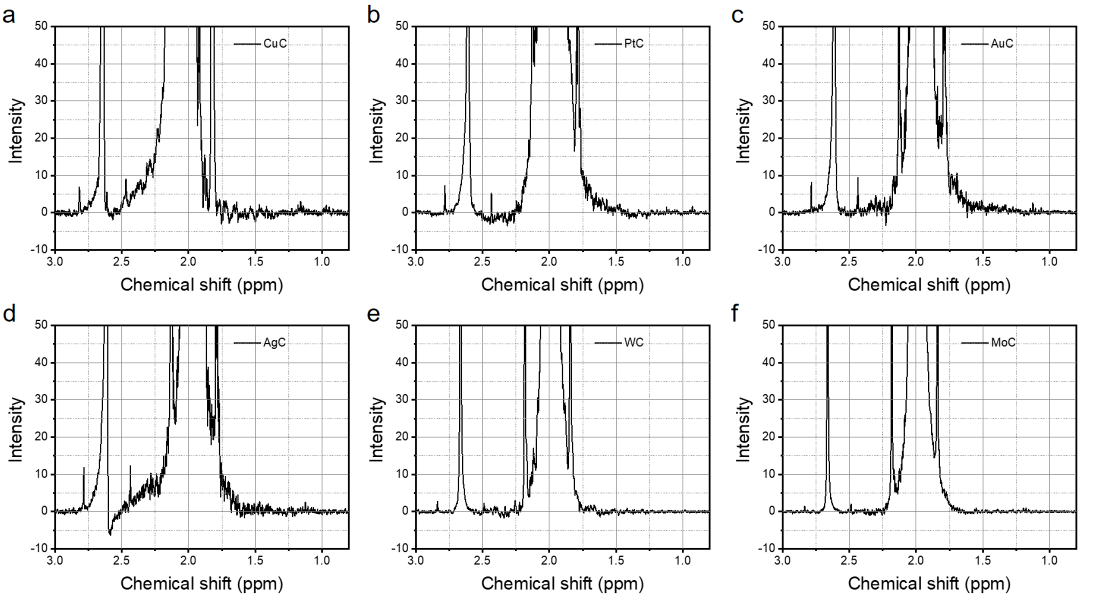

Supplementary Fig. 9. The ^{1}H NMR spectra of electrolyte products by different catalysts. a, Cu/C at -0.29 V, **b,** Pt/C at -0.24 V, **c,** Au/C at -0.324 V, **d,** Ag/C at -0.29 V, **e,** W/C at -0.383 V and **f,** Mo/C at -0.357 V, respectively.

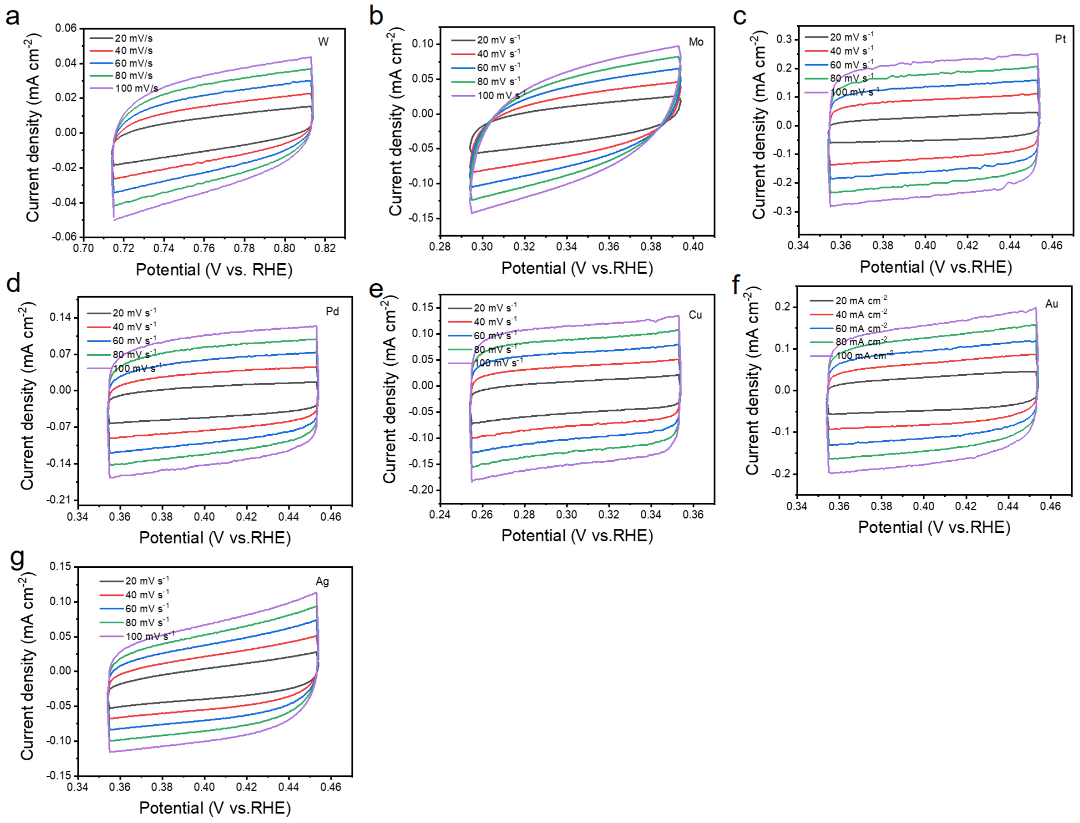

Supplementary Fig. 10. The cyclic voltammetry curves under Ar atmosphere at different scan rates. a, W, **b,** Mo, **c,** Pt, **d,** Pd, **e,** Cu, **f,** Au and **g,** Ag nanoparticles, respectively (without carbon loaded).

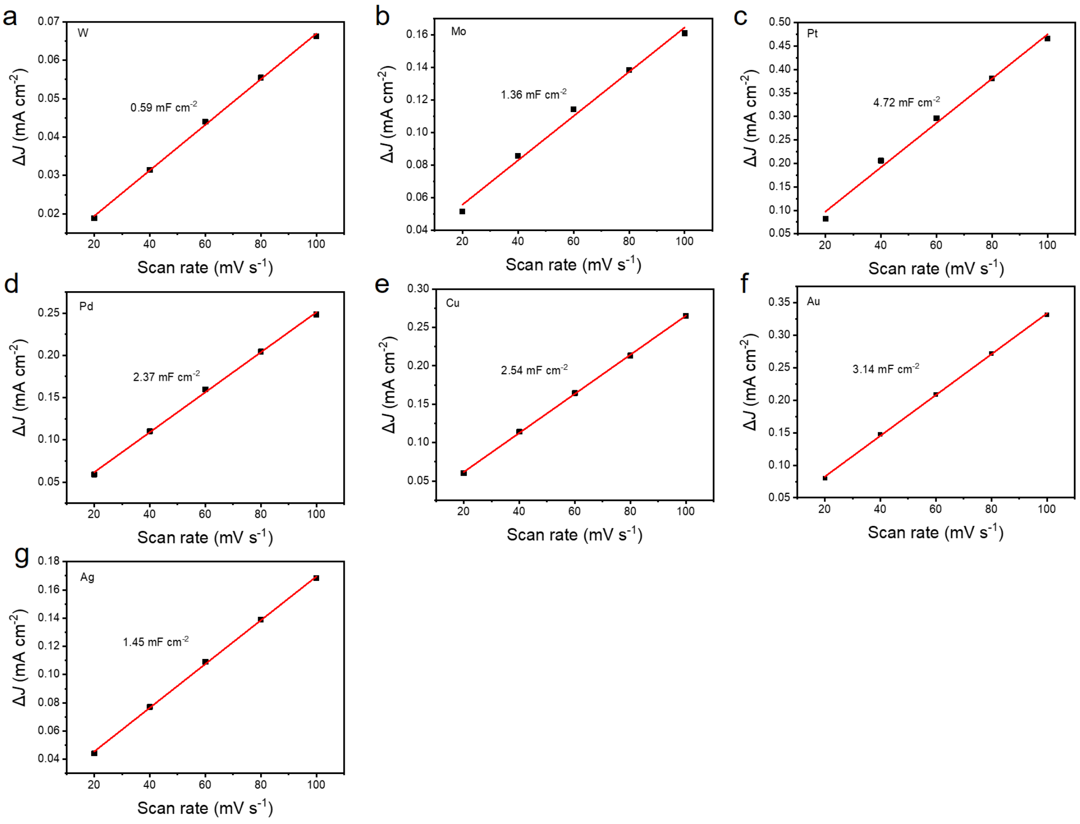

Supplementary Fig. 11. The plots of ΔJ versus scan rates for different catalysts. a, W, **b,** Mo, **c,** Pt, **d,** Pd, **e,** Cu, **f,** Au and **g,** Ag nanoparticles (without carbon loaded).

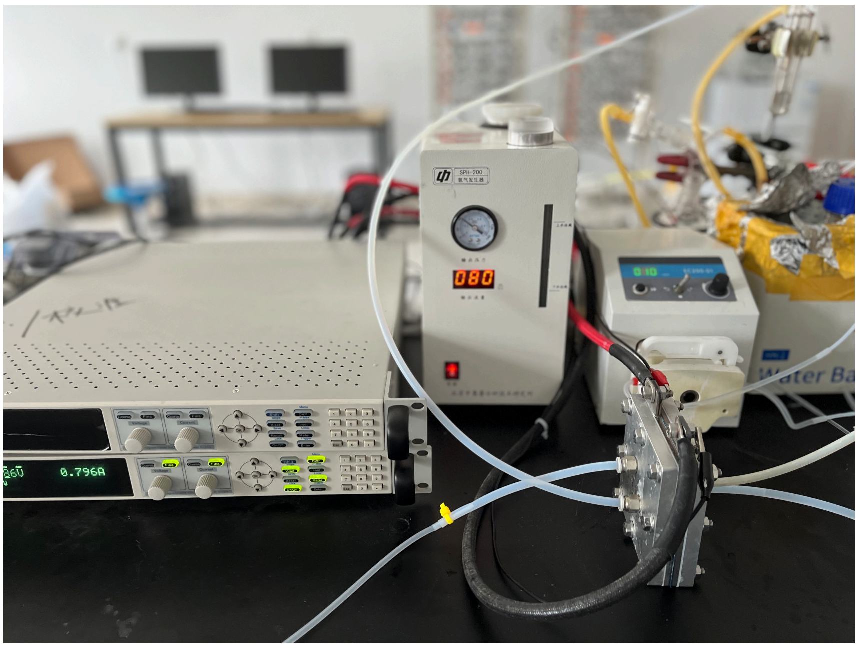

Supplementary Fig. 12. Optical photographs of MEA test system of E-HAN.

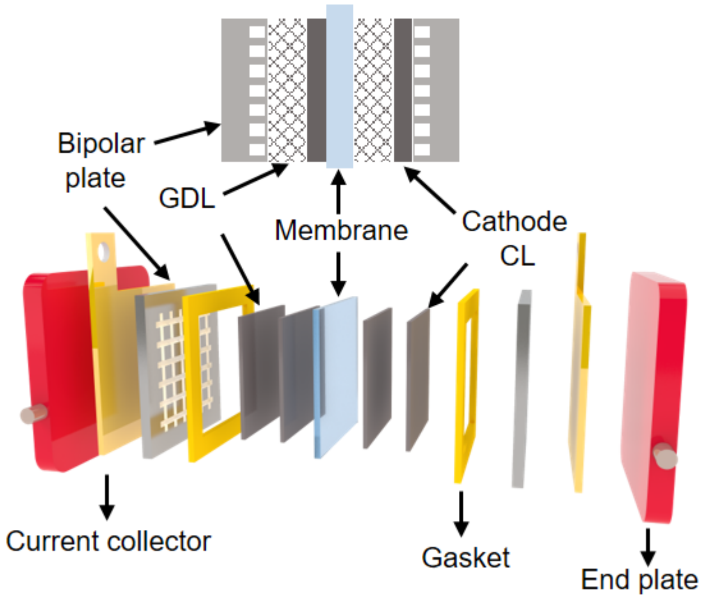

Supplementary Fig. 13. Schematic illustration of the MEA flow cell structure.

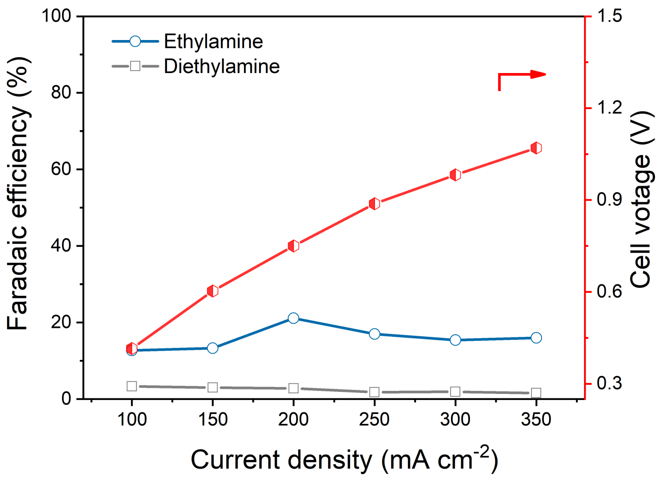

Supplementary Fig. 14. Catalytic performance of the cell prepared by catalyst coated membrane tested in 0.5 M H_2SO_4 containing 8 wt% acetonitrile solution. The cells prepared by catalyst coated membrane (CCM) method was studied to investigate the differences between the microenvironment on PFSA membranes and sulfuric acid. It can be seen from the results that the cells prepared by CCM method exhibited a lower ethylamine faradaic efficiency in 0.5 M H_2SO_4 containing 8 wt% acetonitrile solution compared with catalyst-coated substrate (CCS) method used in this paper. This suggests that the local environment of PFSA might benefit proton transfer and subsequent electrolysis, resulting in enhanced hydrogen evolution reaction (HER)

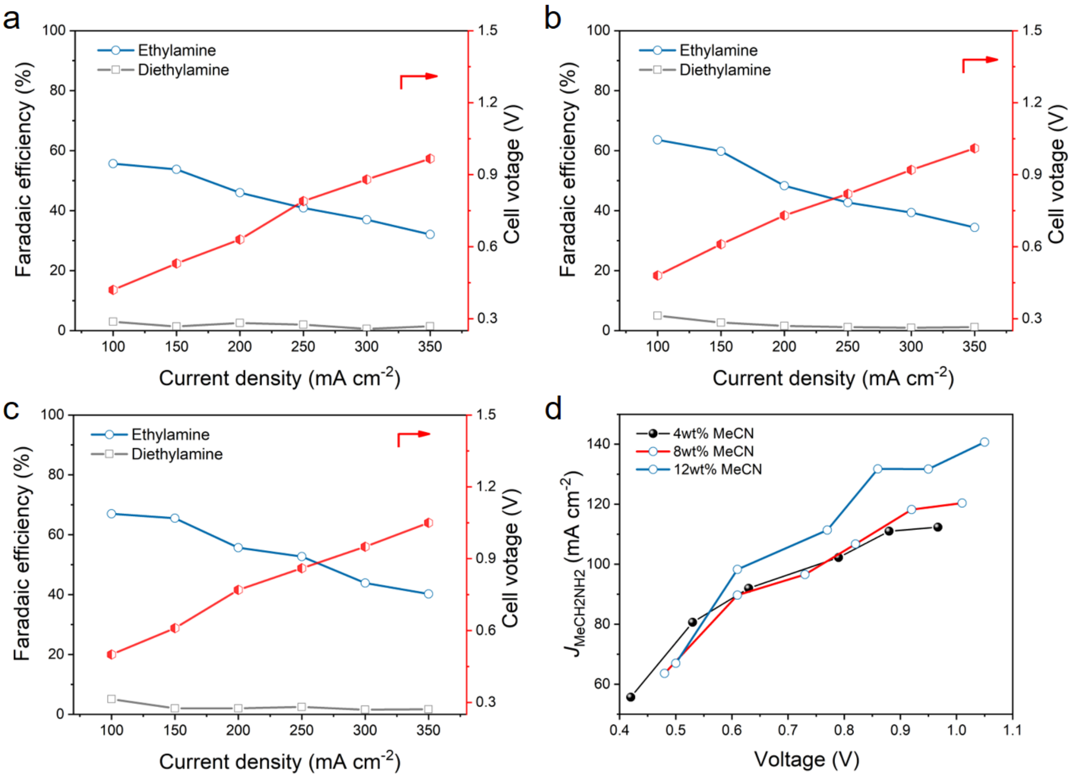

Supplementary Fig. 15. Comparison of acetonitrile electroreduction performance in different concentration of acetonitrile. Ethylamine, diethylamine FE, and corresponding cell voltage versus applied current density on Pd/C testing in 0.5 M H_2SO_4 solution containing 4 wt% acetonitrile (**a**), 0.5 M H_2SO_4 solution containing 8 wt% acetonitrile (**b**) and 0.5 M H_2SO_4 solution containing 12 wt% acetonitrile (**c**). **d,** Ethylamine partial current density under different concentrations of acetonitrile.

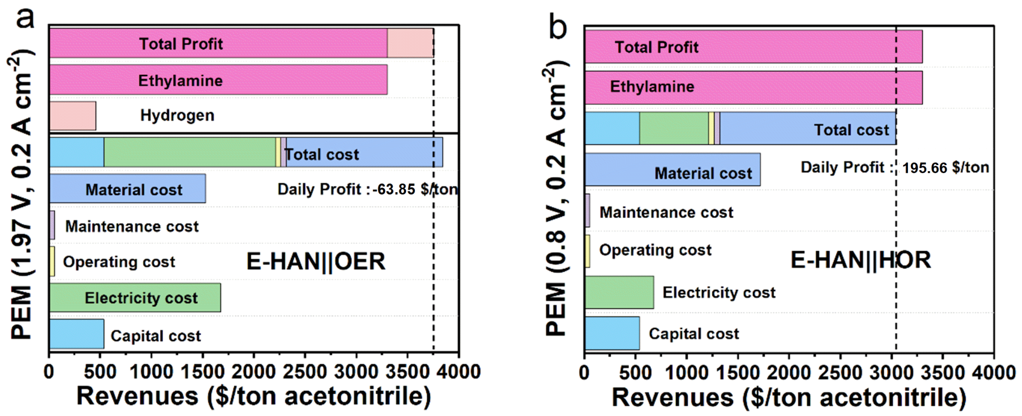

Supplementary Fig. 16. Techno-economic analysis (TEA) of the electrocatalytic hydrogenation of acetonitrile to ethylamine by anodic oxygen evolution or hydrogen oxidation reaction at 200 mA cm^{-2}. a, Oxygen evolution reaction at the anode. **b,** Hydrogen oxidation reaction at the anode. To identify the economic feasibility of renewable electricity powered generation of ethylamine from acetonitrile, we used a simplified techno-economic analysis (TEA) model adapted from the Sargent group reported[1]. The processing capacity of

the plant is 200 tons of acetonitrile per day. **Supplementary Table 1** summarize the price of input chemicals and products. The plant levelized cost model for processing per ton of acetonitrile at a current density of 200 mA cm^{-2} is shown in the **Supplemental Fig. 17**.

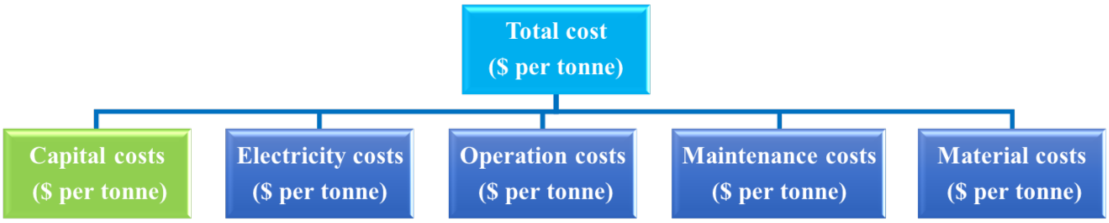

Supplementary Fig. 17. Model used for calculating the techno-economic analysis of generating ethylamine from acetonitrile using electricity. Units are in US$ per ton of acetonitrile.

Calculated assumptions for the TEA of generating EA from AN.

1. The cost of capital include the electrolyzer, catalysts and membranes, and separation equipment. It is assumed that the cost of the electrolyzer is US $10,000/$m^2$ and the lifetime of plant is 10 years. The cost of the catalysts and membranes is 5% of the electrolyzer cost and the separation equipment is 10% of the electrolyzer cost.

2. Assuming a plant operating capacity factor of 0.8, indicating that the plant operates 19.2 h per day

3. Inputs: Acetonitrile, H_2SO_4, water, or H_2. For anode HOR operation, with an electrolyte of 0.5 M H_2SO_4 and 8 wt% acetonitrile, 0.54 tons of sulfuric acid, 10.96 tons of water, and 0.1 tons of H_2 are needed to process 1 ton of acetonitrile, yielding ethylamine (Note: For anode OER operation, with an electrolyte of 0.5 M H_2SO_4 and 8 wt% acetonitrile, 0.54 tons of sulfuric acid, and 10.96 tons of water are needed to process 1 ton of acetonitrile, yielding ethylamine and hydrogen.).

4. During the operation of the electrolyzer, the FEs of ethylamine and H_2 were 40% and 60%, respectively. (Obtained from PEM stability tests).

5. The cost of electricity consists of two parts: the cost of electricity for the electrolyzer and the separation equipment. Assume that the price of electricity is US $0.1/kWh. The cost of electricity for separation equipment is 30% of electrolyzer electricity costs.

6. Both operation and maintenance costs are assumed to be 10% of the capital cost.

Computational process

Note: For anode HOR operation, the consumption of hydrogen is meticulously accounted for.

The precise calculation process is outlined as follows:

1. Electricity costs

The total cost of electricity required to process each ton of acetonitrile：

$$Q = \frac{Mass\ of\ AN\ (g) \times F \times N}{Molar\ mass\ of\ AN(\frac{g}{mol}) \times FE} = \frac{1000000\ \times 96485 \times 4}{41.052 \times 0.4} = 2.35\ \times 10^{10}\ C$$

where Q is the total charge, F is the Faraday constant, and N (4) is the number of transferred electrons.

$$I = \frac{Q}{Time\ in\ a\ day(s) \times Capacity\ factor} = \frac{2.35\ \times 10^{10}}{24 \times 3600 \times 0.8} = 339988.40\ A$$

The operating potential of the electrolyzer is 0.8 V. The power required to maintain the process is calculated as follows：

$$P = \frac{U \times I}{1000} = \frac{0.8\ \times 339988.4}{1000} = 271.99\ kW$$

The daily energy consumption is calculated as：

$$\text{Energy use per day} = \text{P} \times \text{Time in a day} \times \text{Capacity facto}r = 271.99 \times 24 \times 0.8$$

$$= 5222.20\ kWh$$

The daily cost of electricity for the electrolyzer is:

$$\text{Electrolyzer electricity per day} = \frac{\text{Energy use per day} \times \text{Electricity per kWh}}{\text{Mass of ethylene glycol produce}}$$

$$= 5222.2 \times 0.1 = \$522.22$$

The separation cost is 30% of the electrolysis cost and is obtained from the following equation:

$$\text{Separation costs} = 522.22\ \times\ 0.3 = \$156.67$$

Therefore, the total electricity cost is：

$$\text{Total electricity cost} = \text{Electrolyzer electricity} + \text{Separation costs}$$

$$= 522.22 + 156.67 = \$678.89$$

2. Capital cost

a. Electrolyzer cost. Based on the operating current density (200 mA cm^{-2}), the obtained electrolyzer area is calculated based on the following formula：

$$\text{Area of electrolyse} = \frac{339988.4\ A}{0.2\ A\ cm^{-2}} = 170.00\ m^2$$

Therefore, the cost of the electrolyzer is calculated as follows:

$$\text{Cost of electrolyser} = 170.00 \times 10000 = \$1700000.00$$

b. Total cost of catalyst and membrane

$$\text{Cost of catalyst and membrane} = 1700000 \times 0.05 = \$\,85000.00$$

c. Capital cost of separation equipment

$$\text{Cost of separations equipment} = 1700000 \times 0.1 = \$\,170000.00$$

Therefore, the cost of capital is:

$$Capital\ costs = \frac{1700000 + 85000 + 170000}{365 \times 10} = \$\,535.62$$

3. Material costs

The cost of the input chemicals requires acetonitrile (1 tons), H_2SO_4 (0.54 tons), water (10.96 tons) and hydrogen.

The mass of hydrogen required for converting 1 ton of acetonitrile is:

$$Hydrogen\ mass = \frac{Q \times \text{Molar mass of hydrogen}}{N \times \text{F}} = \frac{2.35 \times 10^{10} \times 2}{2 \times 96485} = 0.24\ ton$$

$$\begin{aligned}\text{Input costs of chemicals} \\ &= \text{Cost of AN} \times \text{Mass of AN} + \text{Cost of } H_2SO_4 \times \text{Mass of } H_2SO_4 \\ &+ \text{Cost of water} \times \text{Mass of water} \\ &+ \text{Input costs of Hydrogen} \times \text{Hydrogen mass obtained} = \$1982.71\end{aligned}$$

4. Operating costs are 10% of the capital costs and is calculated as:

$$\text{Operating costs} = 535.62 \times 0.1 = \$\,53.56$$

5. Maintenance costs are 10% of the capital cost and is calculated as:

$$\text{Maintenance costs} = 535.62 \times 0.1 = \$\,53.56$$

Therefore, the total costs is obtained by adding up the five components:

$$\begin{aligned}\text{Total costs} &= \text{Input chemicals} + \text{Electricity cost} + \text{Capital cost} + \text{Operating cost} \\ &+ \text{Maintenance cost} = \$3304.34\end{aligned}$$

Profit

The products of the electrochemical acetonitrile hydrogenation process are ethylamine and hydrogen. Of these, 0.35 tons of ethylamine are obtained per ton of acetonitrile feedstock. Therefore, the value of the product is calculated as:

$$\text{Ethylamine product value} = \text{Ethylamine cost} \times \text{Ethylamine mass obtaine} = \$3500.00$$

$$\text{Total profit per ton of AN} = \text{Ethylamine profit} = \$3500.00$$

Therefore, daily profit per ton AN can be calculated as:

$$\text{Daily Profit} = \text{Total Profit} - \text{Total Cost} = \$195.66/\text{ton}$$

Note: For anode OER operation, the products are ethylamine and hydrogen. It is noted that the sole cost variation during calculation is the electrical expense, while all other costs remain consistent with prior computations. The specific calculation is described below:

1. Electricity costs

The total cost of electricity required to process each ton of acetonitrile：

$$Q = \frac{Mass\ of\ AN\ (g) \times F \times N}{Molar\ mass\ of\ AN(\frac{g}{mol}) \times FE} = \frac{1000000\ \times 96485 \times 4}{41.052 \times 0.4} = 2.35\ \times 10^{10}\ C$$

where Q is the total charge, F is the Faraday constant, and N (4) is the number of transferred electrons.

$$I = \frac{Q}{Time\ in\ a\ day(s) \times Capacity\ factor} = \frac{2.35\ \times 10^{10}}{24 \times 3600 \times 0.8} = 339988.40\ A$$

The operating potential of the electrolyzer is 1.97 V. The power required to maintain the process is calculated as follows：

$$P = \frac{U \times I}{1000} = \frac{1.97\ \times 339988.4}{1000} = 669.78\ kW$$

The daily energy consumption is calculated as：

$$\text{Energy use per day} = \text{P} \times \text{Time in a day} \times \text{Capacity facto}r = 669.78 \times 24 \times 0.8$$

$$= 12859.78\ kWh$$

The daily cost of electricity for the electrolyzer is:

$$\text{Electrolyzer electricity per day} = \frac{\text{Energy use per day} \times \text{Electricity per kWh}}{\text{Mass of ethylene glycol produce}}$$

$$= 12859.78 \times 0.1 = \$1286.00$$

The separation cost is 30% of the electrolysis cost and is obtained from the following equation:

$$\text{Separation costs} = 1286.0 \times 0.3 = \$385.80$$

Therefore, the total electricity cost is：

$$\text{Total electricity cost} = \text{Electrolyzer electricity} + \text{Separation costs}$$

$$= 1286.0 + 385.8 = \$1671.80$$

Therefore, the total costs is obtained by adding up the five components：

$$\text{Total costs} = \text{Input chemicals} + \text{Electricity cost} + \text{Capital cost} + \text{Operating cost} + \text{Maintenance cost} = \$3841.25$$

Profit

The products of the electrochemical acetonitrile hydrogenation process are ethylamine and hydrogen. Of these, 0.35 tons of ethylamine are obtained per ton of acetonitrile feedstock. Therefore, the value of the product is calculated as：

$$\text{Ethylamine product value} = \text{Ethylamine cost} \times \text{Ethylamine mass obtaine} = \$3500.00$$

$$\text{Hydrogen profit} = \text{Hydrogen cost} \times \text{Hydrogen mass obtained} = \$456.00$$

The Faraday efficiency of hydrogen is 60%, so the mass of hydrogen is:

$$\text{Hydrogen mass} = \frac{Q \times Molar\ mass\ of\ hydrogen}{N \times F} = 240\ \text{kg}$$

$$\text{Hydrogen profit} = \text{Hydrogen cost} \times \text{Hydrogen mass obtained} = 277.4\ \$/\text{ton}$$

Therefore, the product value can be calculated as:

$$\text{Total profit per ton of AN} = \text{Ethylamine profit} + \text{Hydrogen profit} = \$3777.4$$

Therefore, daily profit per ton AN can be calculated as:

$$\text{Daily Profit} = \text{Total Profit} - \text{Total Cost} = -\$63.85$$

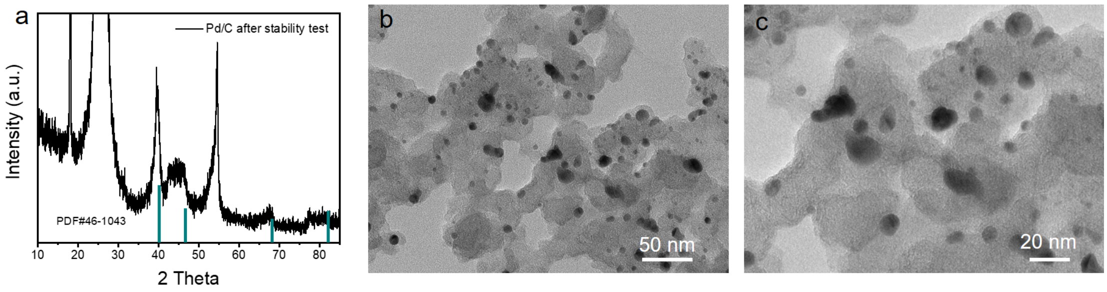

Supplementary Fig. 18. Post reaction characterizations of XRD and TEM. a, PXRD patterns and **b, c,** TEM images of Pd/C catalysts after the 20-hour stability test in 0.5 M H_2SO_4 containing 8 wt% acetonitrile as electrolyte at 200 mA cm^{-2}. The a.u. stands for arbitrary units.

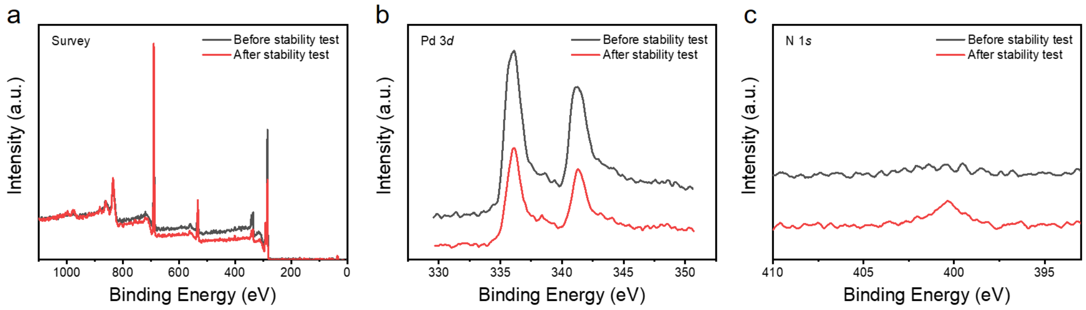

Supplementary Fig. 19. Post reaction characterization of XPS. XPS survey spectra (**a**), Pd 3*d* (**b**) and N 1*s* (**c**) spectra of Pd/C catalysts before and after the 20-hour stability test in 0.5 M H_2SO_4 containing 8 wt% acetonitrile as electrolyte at 200 mA cm^{-2}. The a.u. stands for arbitrary units.

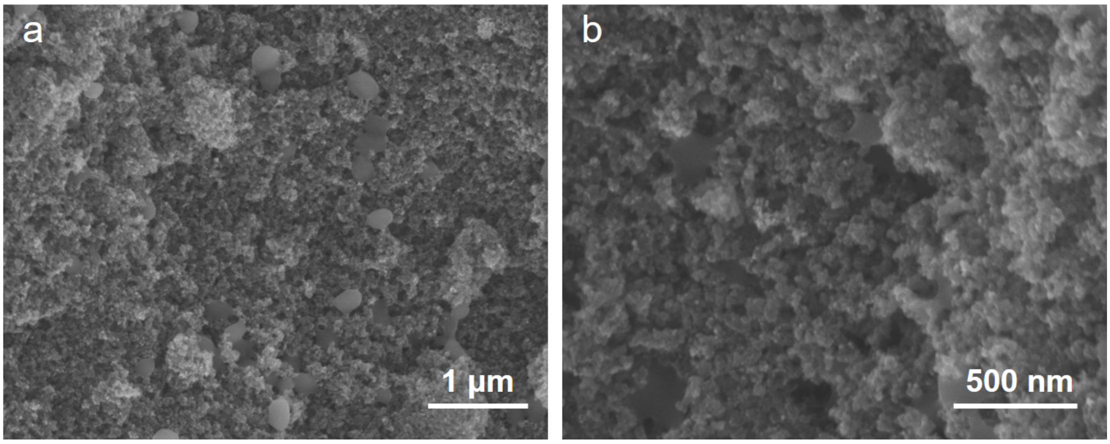

Supplementary Fig. 20. SEM characterization. a, b, SEM images of Pd/C on gas diffusion layer.

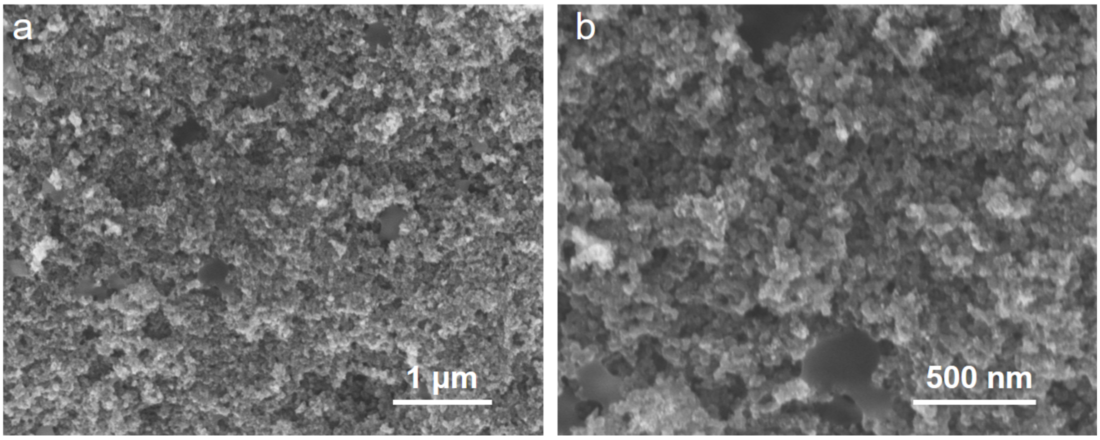

Supplementary Fig. 21. SEM characterization. a, b, SEM images of Pd/C catalysts after the 20-hour stability test in 8 wt% acetonitrile 0.5 M H_2SO_4 at 200 mA cm^{-2}.

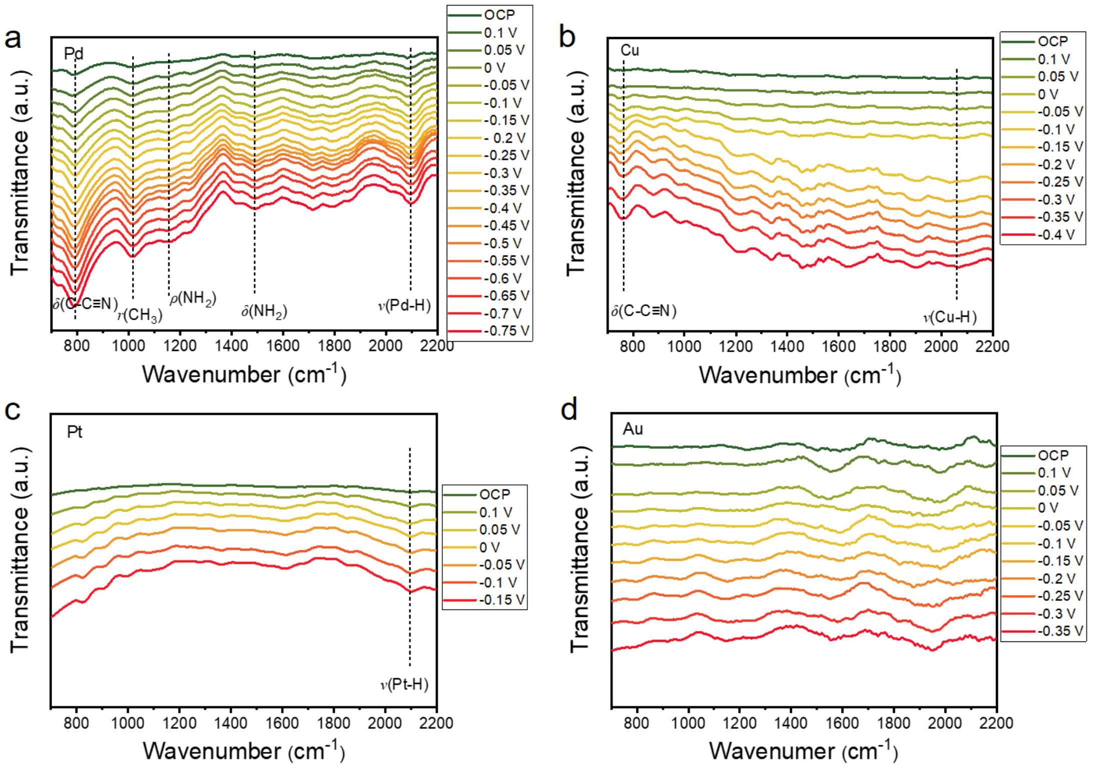

Supplementary Fig. 22. The operando SR-FTIR characterization. SR-FTIR spectra on Pd (**a**), Cu (**b**), Pt (**c**) and Au (**d**) at potentials vs. RHE in 0.5 M H_2SO_4 containing 8 wt% acetonitrile as electrolyte. The a.u. stands for arbitrary units.

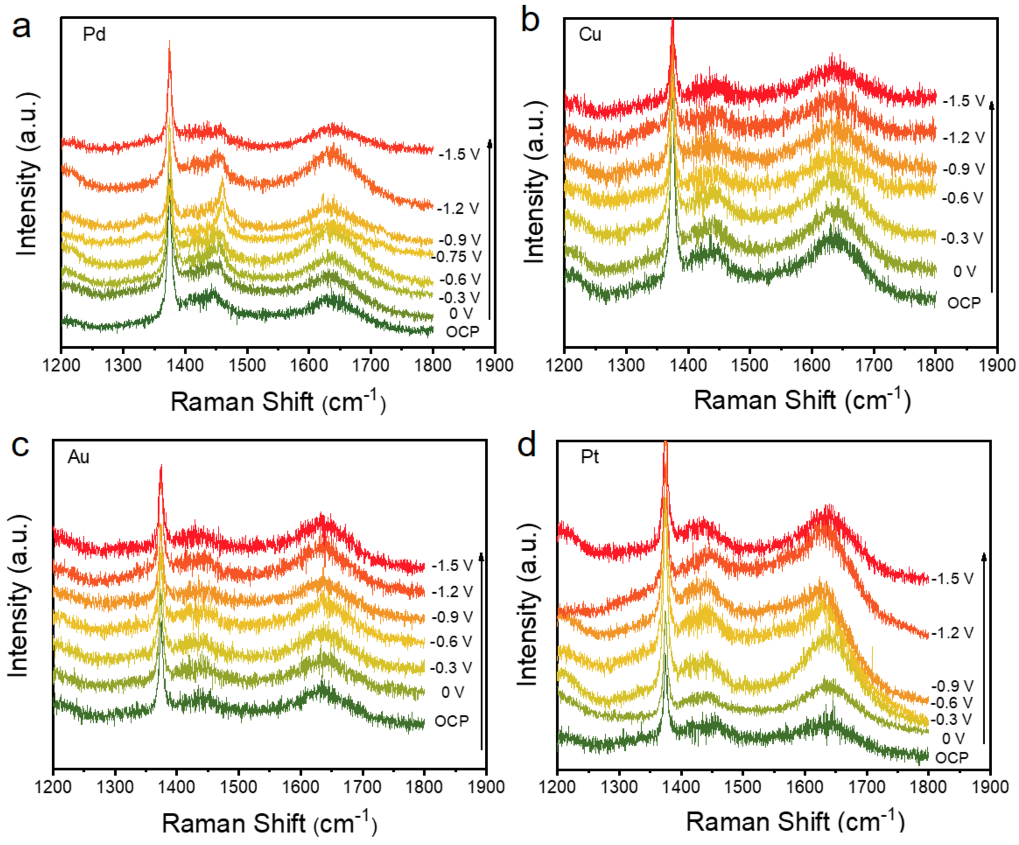

Supplementary Fig. 23. The operando Raman characterization. Raman spectra of E-HAN on a Pd (**a**), Cu (**b**), Au (**c**) and Pt (**d**) in 0.5 M H_2SO_4 containing 8 wt% acetonitrile as electrolyte. The a.u. stands for arbitrary units.

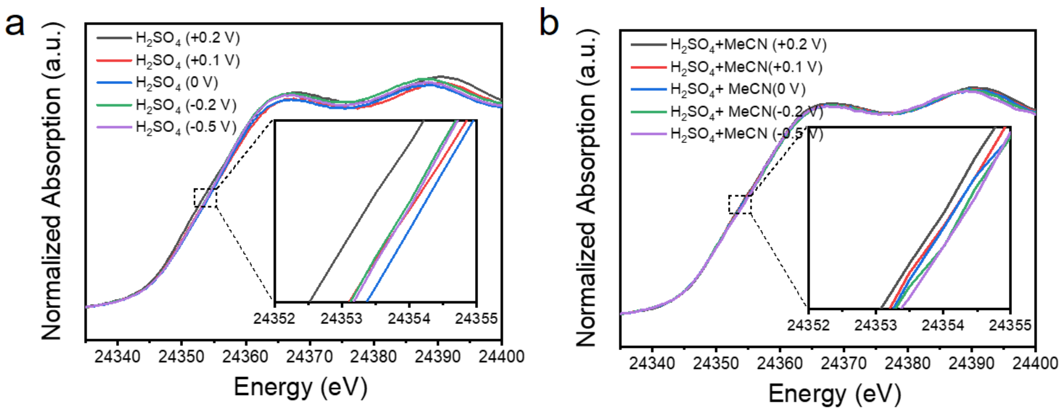

Supplementary Fig. 24. The operando XANES characterization. Potential dependence of operando Pd K-edge XANES spectra of the catalyst in 0.5 M H_2SO_4 solution (**a**) and after the addition of 8 wt% acetonitrile (**b**). The a.u. stands for arbitrary units.

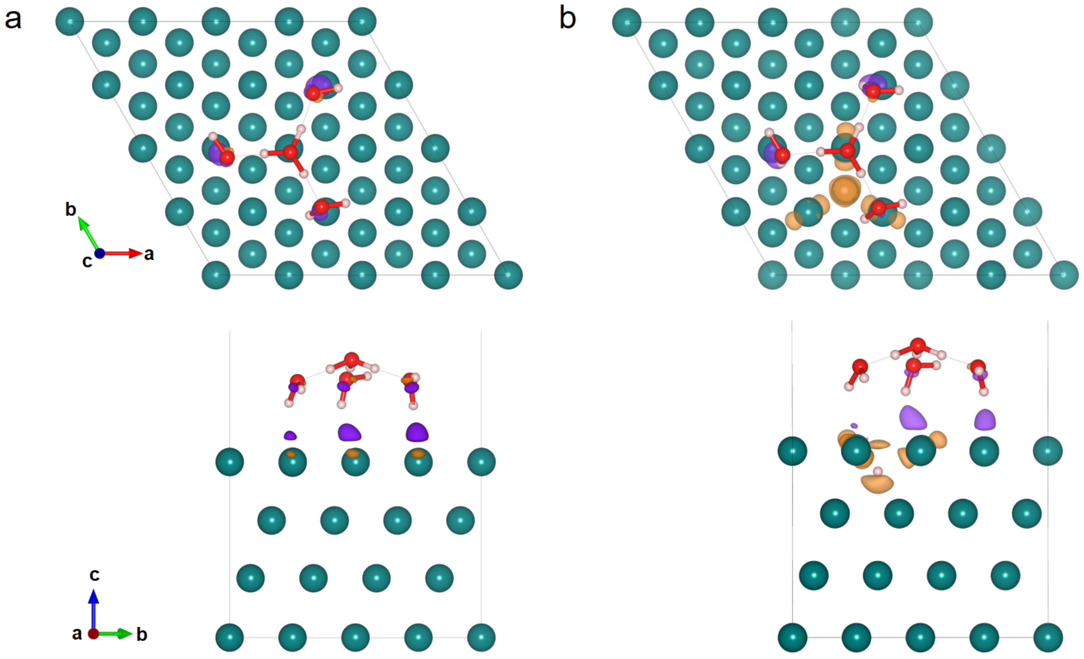

Supplementary Fig. 25. The top-view and side-view differential electron density of H_9O_4 adsorbed on Pd (111) (**a**) and PdH_x (111) (**b**). Yellow and purple colours represent charge depletion and accumulation, respectively, with an iso-surface value of 0.005 eA^{-3}.

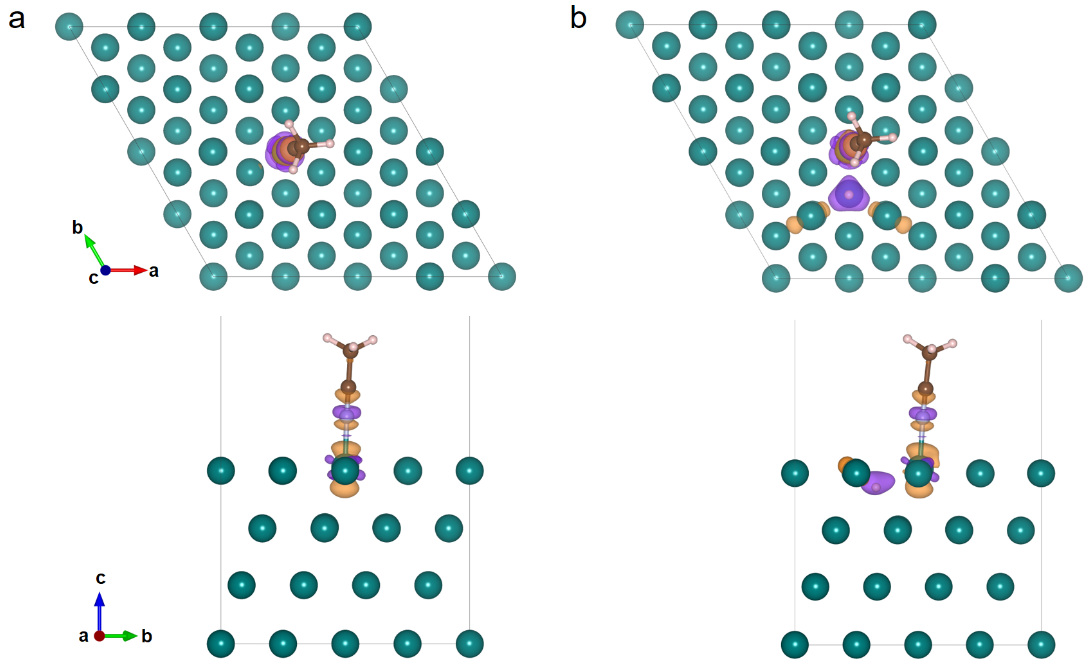

Supplementary Fig. 26. The top-view and side-view differential electron density of MeCN adsorbed on Pd (111) (**a**) and PdH_x (111) (**b**). Yellow and purple colours represent charge depletion and accumulation, respectively, with an iso-surface value of 0.005 eÅ^{-3}.

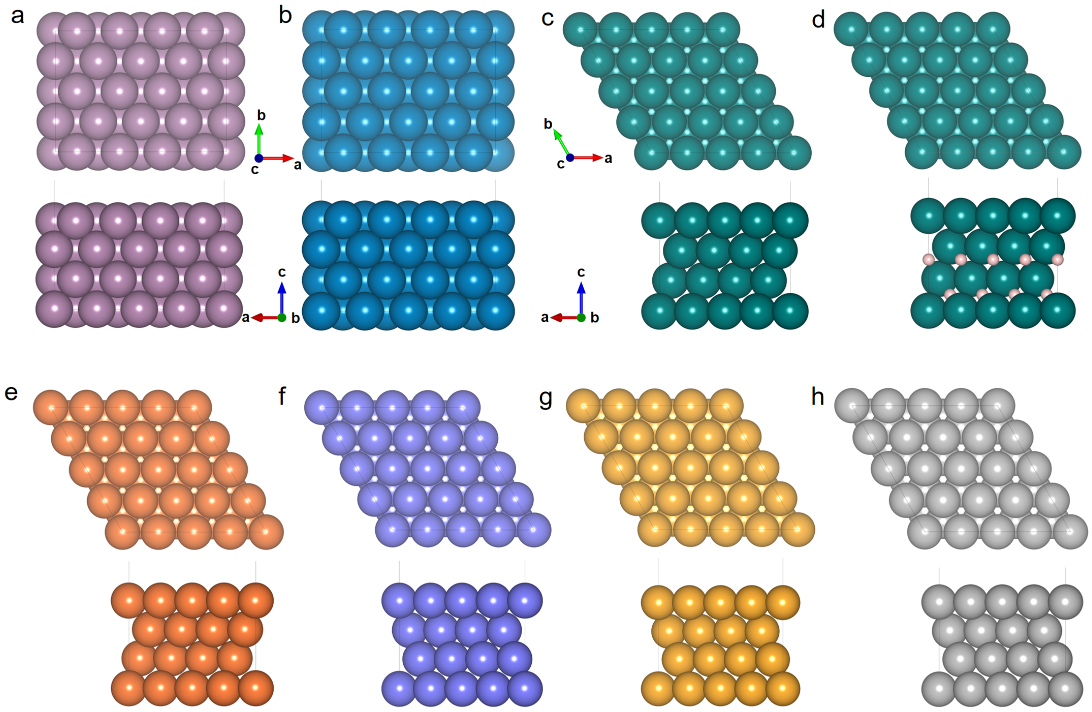

Supplementary Fig. 27. Calculation models for different metal surface. a, Mo (110), **b,** Mo (110), **c,** Pd (111), **d,** PdH_x (111), **e,** Cu (111), **f,** Pt (111), **g,** Au (111) and **h,** Ag (111).

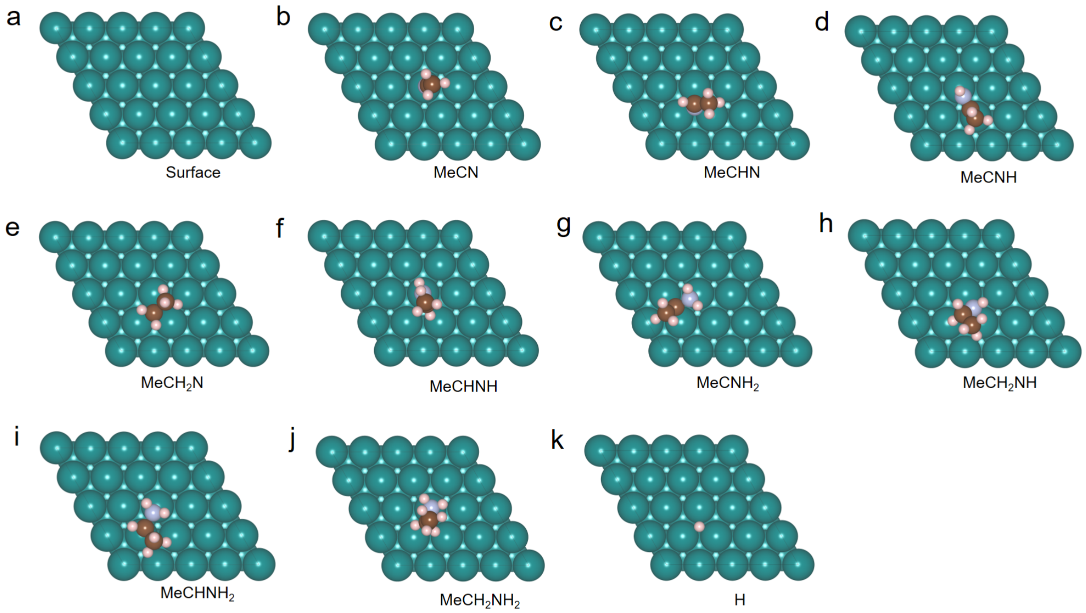

Supplementary Fig. 28. DFT optimized geometries on Pd (111) surface with *H coverage of 0-16ML. Top image views of surface (**a**), *MeCN (**b**), *MeCHN (**c**), *MeCNH (**d**), *$MeCH_2N$ (**e**), *MeCHNH (**f**), *$MeCNH_2$ (**g**), *$MeCH_2NH$ (**h**), *$MeCHNH_2$ (**i**), *$CH_3CH_2NH_2$ (**j**) and *H (**k**).

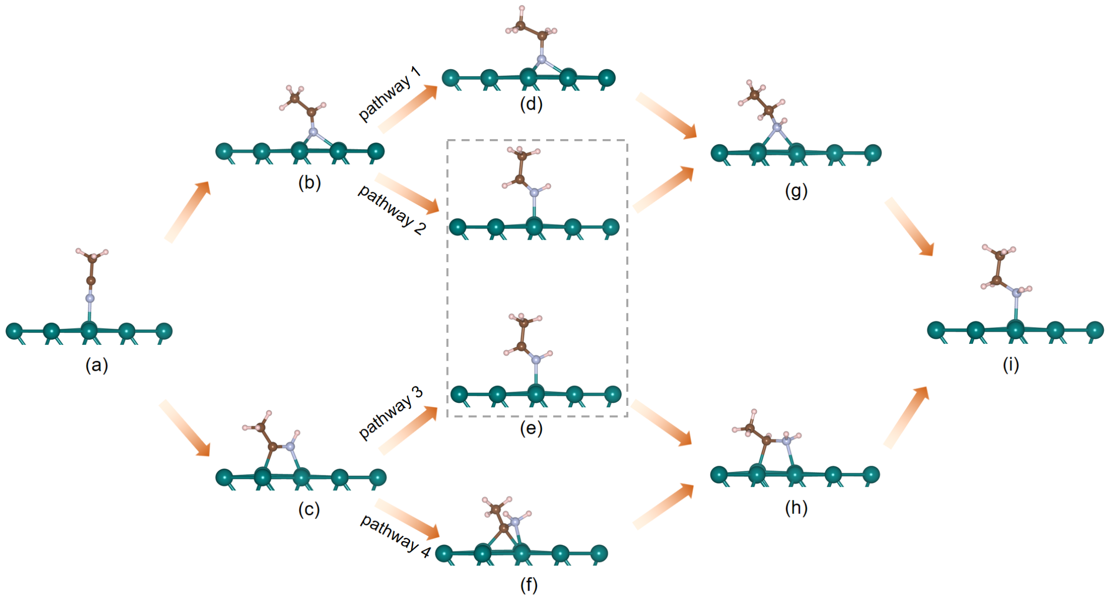

Supplementary Fig. 29. Schematic of six potential pathways for CH_3CN reduction reaction. a, *MeCN, **b,** *MeCHN, **c,** *MeCNH, **d,** *$MeCH_2N$, **e,** *MeCHNH, **f,** *$MeCNH_2$, **g,** *$MeCH_2NH$, **h,** *$MeCHNH_2$ and **i,** *$MeCH_2NH_2$.

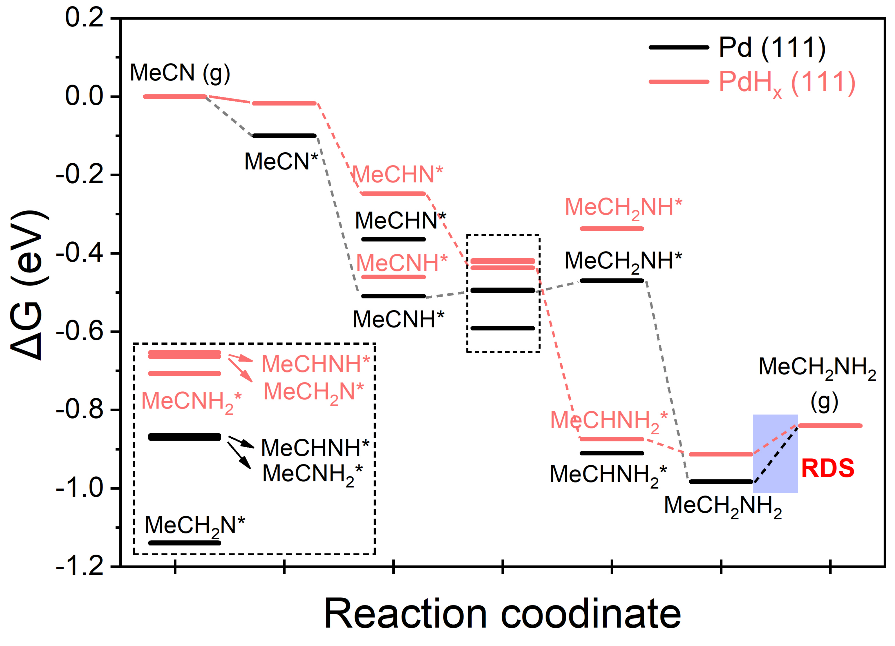

Supplementary Fig. 30. Free energy diagrams on Pd (111) and PdH$_x$ (111) surface at an applied potential U = 0 V.

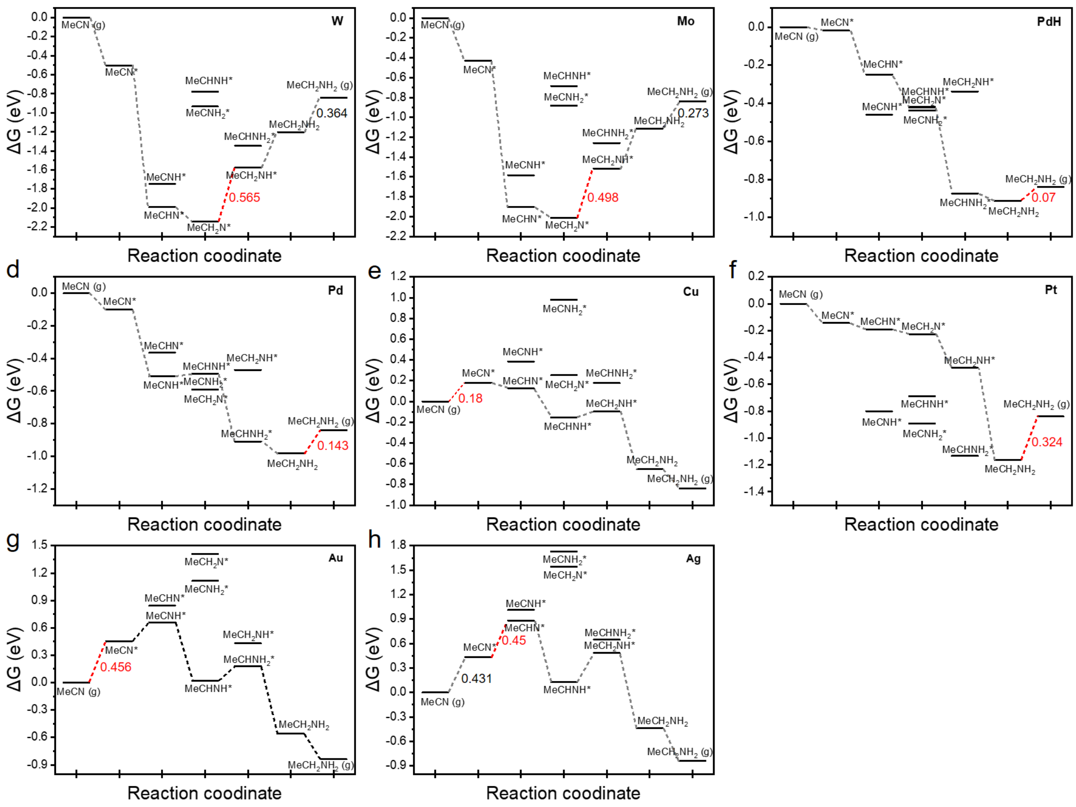

Supplementary Fig. 31. Free energy diagrams for MeCN (g) electroreduction to $MeCH_2NH_2$ (g) on different catalyst surfaces. a, W (110), **b,** Mo (110), **c,** Pd (111), **d,** PdH$_x$ (111), **e,** Cu (111), **f,** Pt (111), **g,** Au (111) and **h,** Ag (111) at an applied potential U = 0 V.

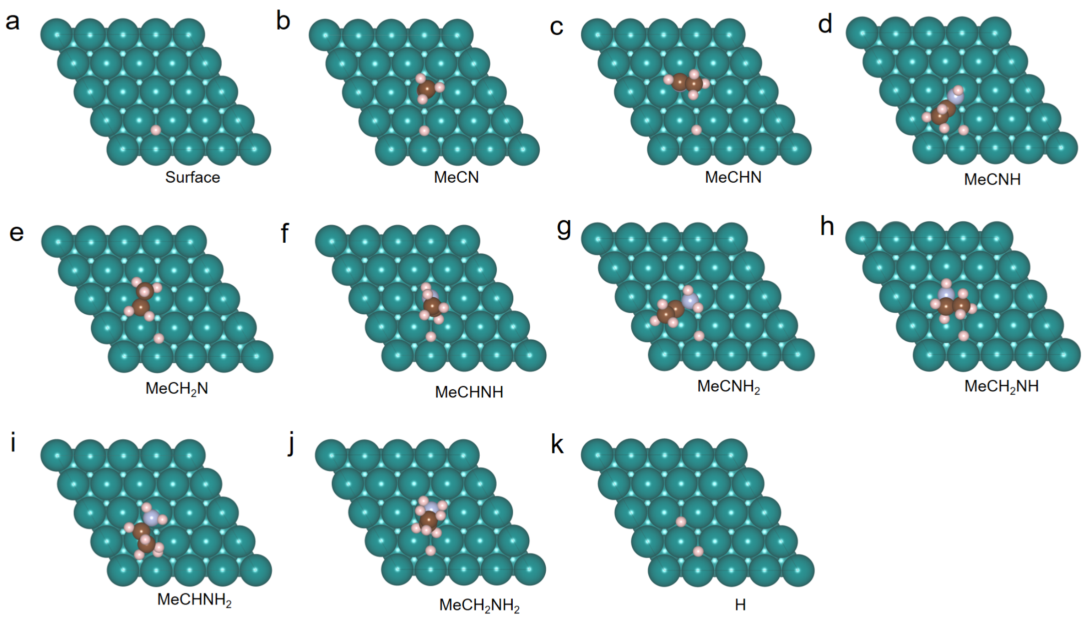

Supplementary Fig. 32. DFT optimized geometries on Pd (111) surface with *H coverage of 1-16ML. Top image views of surface (**a**), *MeCN (**b**), *MeCHN (**c**), *MeCNH (**d**), *$MeCH_2N$ (**e**), *MeCHNH (**f**), *$MeCNH_2$ (**g**), *$MeCH_2NH$ (**h**), *$MeCHNH_2$ (**i**), *$CH_3CH_2NH_2$ (**j**) and *H (**k**).

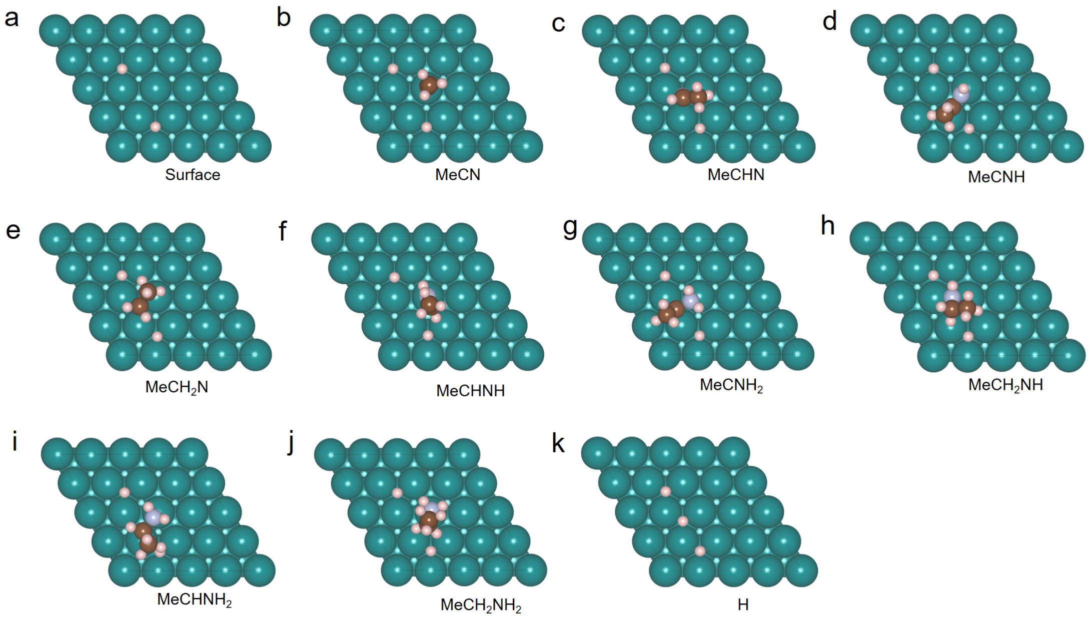

Supplementary Fig. 33. DFT optimized geometries on Pd (111) surface with *H coverage of 2-16ML. Top image views of surface (**a**), *MeCN (**b**), *MeCHN (**c**), *MeCNH (**d**), *$MeCH_2N$ (**e**), *MeCHNH (**f**), *$MeCNH_2$ (**g**), *$MeCH_2NH$ (**h**), *$MeCHNH_2$ (**i**), *$CH_3CH_2NH_2$ (**j**) and *H (**k**).

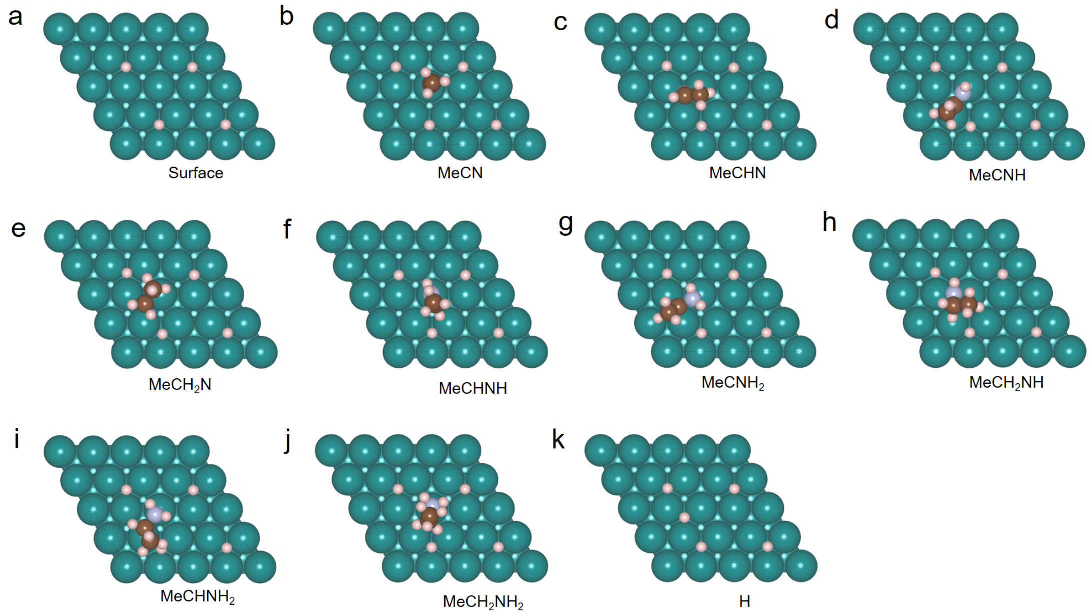

Supplementary Fig. 34. DFT optimized geometries on Pd (111) surface with *H coverage of 4-16ML. Top image views of surface (**a**), *MeCN (**b**), *MeCHN (**c**), *MeCNH (**d**), *MeCH$_2$N (**e**), *MeCHNH (**f**), *MeCNH$_2$ (**g**), *MeCH$_2$NH (**h**), *MeCHNH$_2$ (**i**), *CH$_3$CH$_2$NH$_2$ (**j**) and *H (**k**).

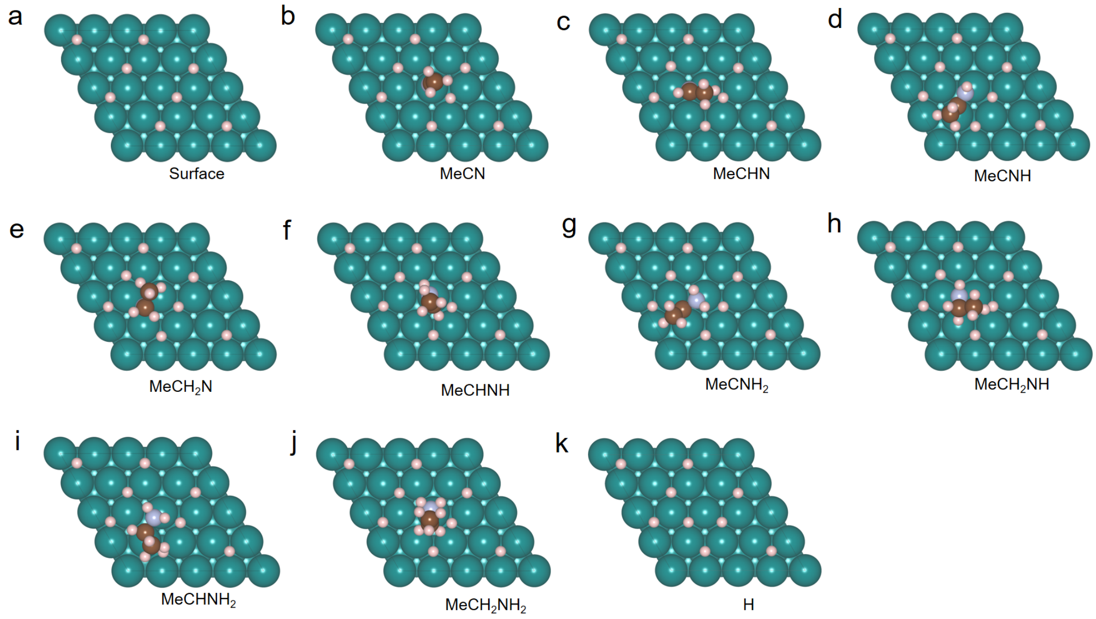

Supplementary Fig. 35. DFT optimized geometries on Pd (111) surface with *H coverage of 8-16ML. Top image views of surface (**a**), *MeCN (**b**), *MeCHN (**c**), *MeCNH (**d**), *MeCH$_2$N (**e**), *MeCHNH (**f**), *MeCNH$_2$ (**g**), *MeCH$_2$NH (**h**), *MeCHNH$_2$ (**i**), *CH$_3$CH$_2$NH$_2$ (**j**) and *H (**k**).

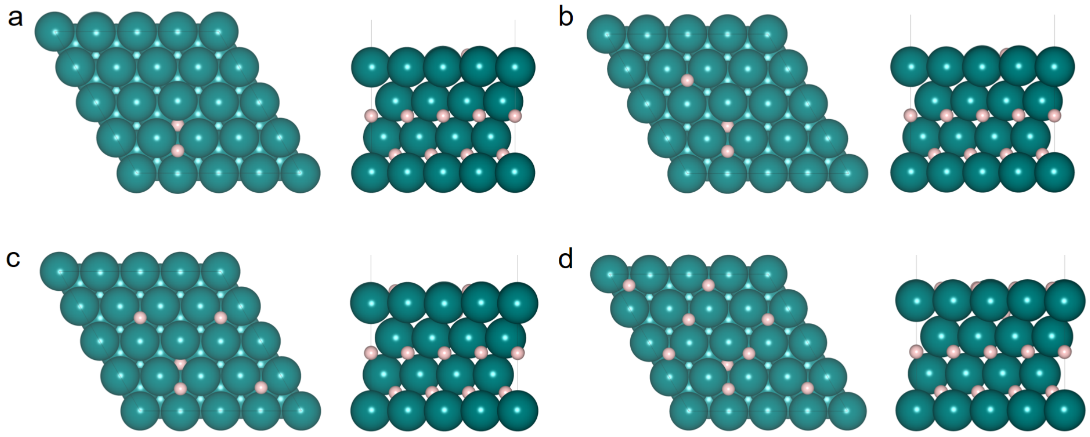

Supplementary Fig. 36. DFT optimized geometries on PdH$_x$ (111) surface with different *H coverages. a, 1-16 ML, **b,** 2-16 ML, **c,** 4-16 ML and **d,** 8-16 ML. The Pd-terminated PdH$_x$ (111) was modeled using the NaCl (L$_{12}$) crystal structure. The PdH$_x$ (111) surfaces were modeled with 4×4 surface slabs consisting of two bilayers and two single layer (a bilayer contains a unit of one Pd layer and one H layer). In addition, because hydrogen could also be present in the subsurface layer during the reaction, we considered a hydrogen doping in the subsurface layer in these models.

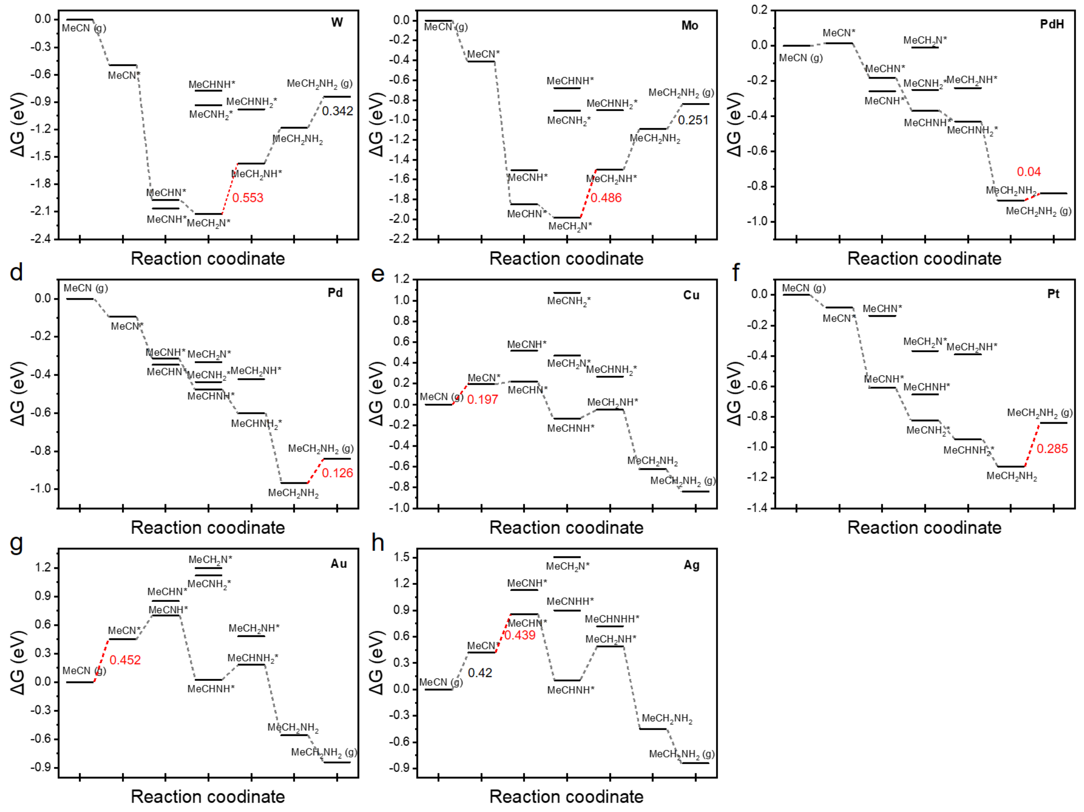

Supplementary Fig. 37. Free energy diagrams for different catalyst surfaces with *H coverage of 1-16ML. a, W (110), **b,** Mo (110), **c,** Pd (111), **d,** PdH$_x$ (111), **e,** Cu (111), **f,** Pt (111), **g,** Au (111) and **h,** Ag (111) at an applied potential U = 0 V.

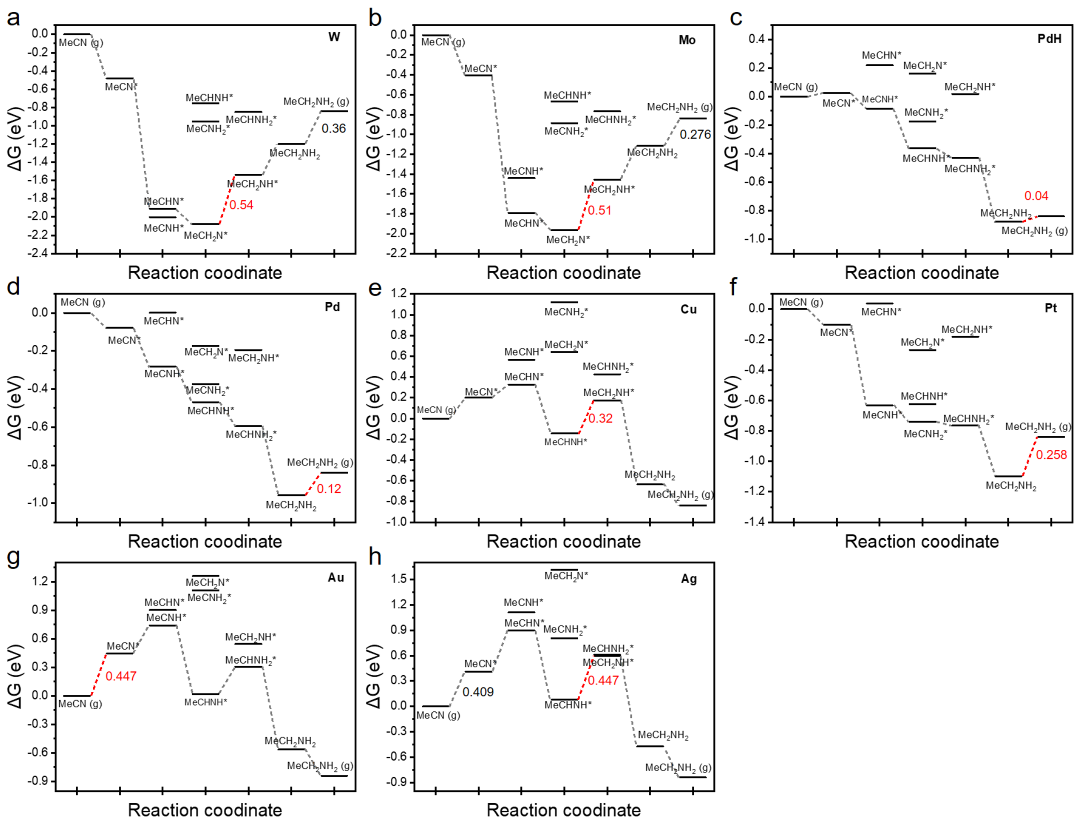

Supplementary Fig. 38. Free energy diagrams for different catalyst surfaces with *H coverage of 2-16ML. a, W (110), **b,** Mo (110), **c,** Pd (111), **d,** PdH_x (111), **e,** Cu (111), **f,** Pt (111), **g,** Au (111) and **h,** Ag (111) at an applied potential U = 0 V.

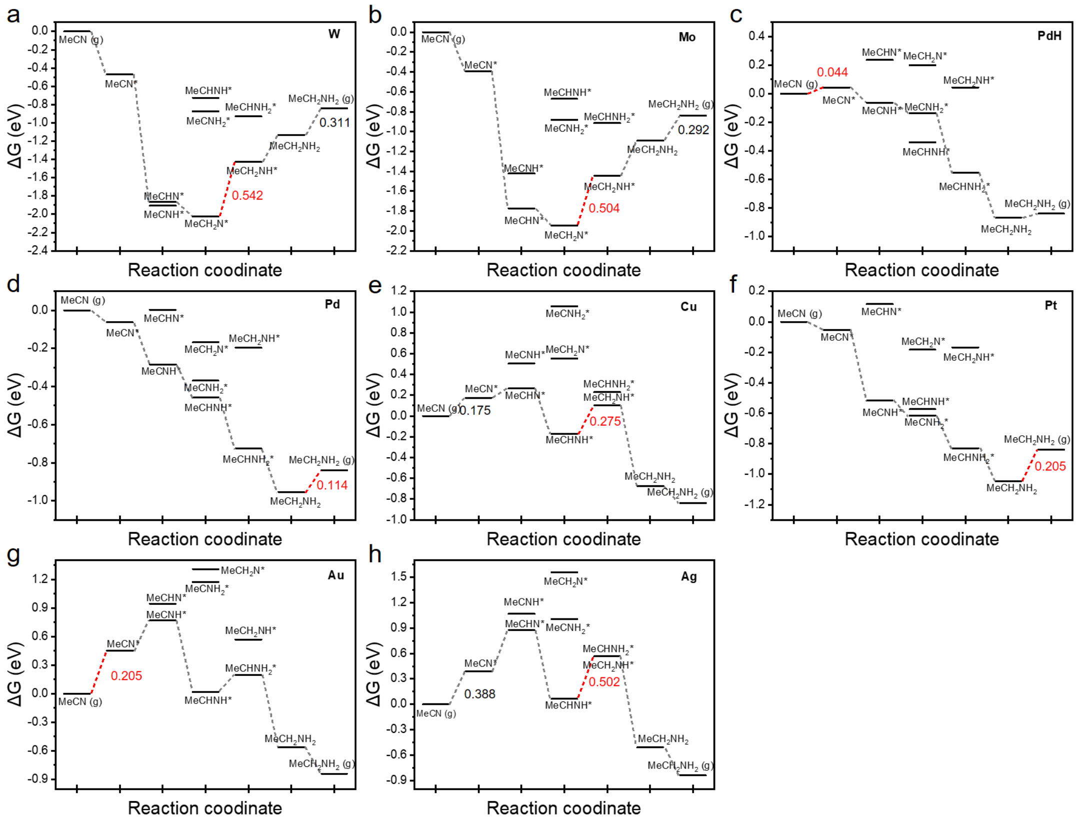

Supplementary Fig. 39. Free energy diagrams for different catalyst surfaces with *H coverage of 4-16ML. a, W (110), **b,** Mo (110), **c,** Pd (111), **d,** PdH_x (111), **e,** Cu (111), **f,** Pt (111), **g,** Au (111) and **h,** Ag (111) at an applied potential U = 0 V.

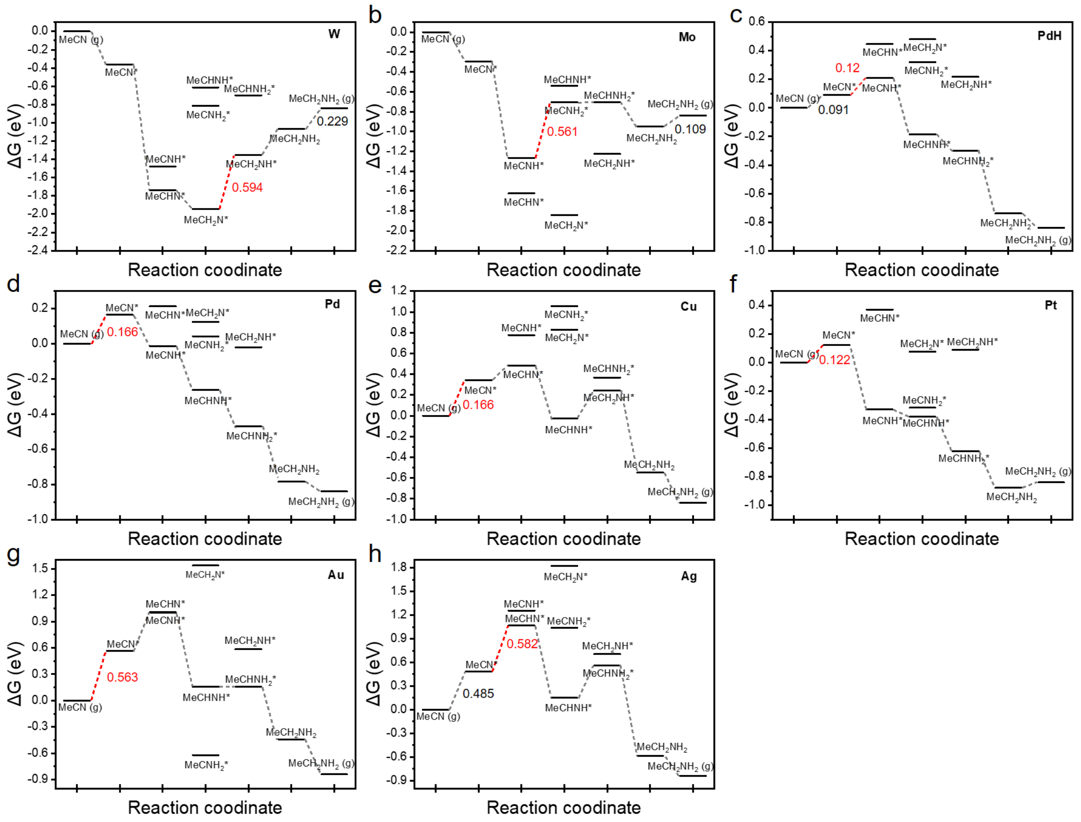

Supplementary Fig. 40. Free energy diagrams for different catalyst surfaces with *H coverage of 8-16ML. a, W (110), **b,** Mo (110), **c,** Pd (111), **d,** PdH_x (111), **e,** Cu (111), **f,** Pt (111), **g,** Au (111) and **h,** Ag (111) at an applied potential U = 0 V.

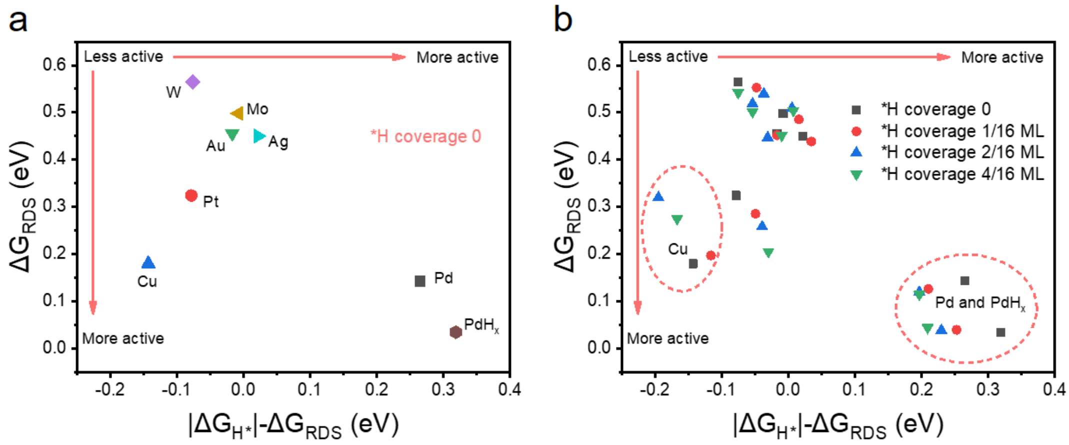

Supplementary Fig. 41. Relationship between $|\Delta G_{H*}|$-ΔG_{RDS} and ΔG_{RDS}. a, *H coverage of 0 ML and **b,** under various *H coverages. In order to more fully understand the activity trend of different metals under acidic conditions, the adsorption energy of hydrogen (ΔG_{H*}) should also be considered. The large $|\Delta G_{H*}|$ and small ΔG_{RDS} indicate that catalyst surface is favorable for acetonitrile hydrogenation but not for the hydrogen

evolution reaction, thus, $|\Delta G_{H*}|-\Delta G_{RDS}$ can be used as the other descriptive factor. With $|\Delta G_{H*}|-\Delta G_{RDS}$ as the horizontal coordinate and ΔG_{RDS} as the vertical coordinate, it can be determined that the catalyst show better performance as its coordinate is close to the lower right. Among these metals, Pd-based catalysts have the best catalytic performance with various *H coverages, which is significantly higher than that of Cu.

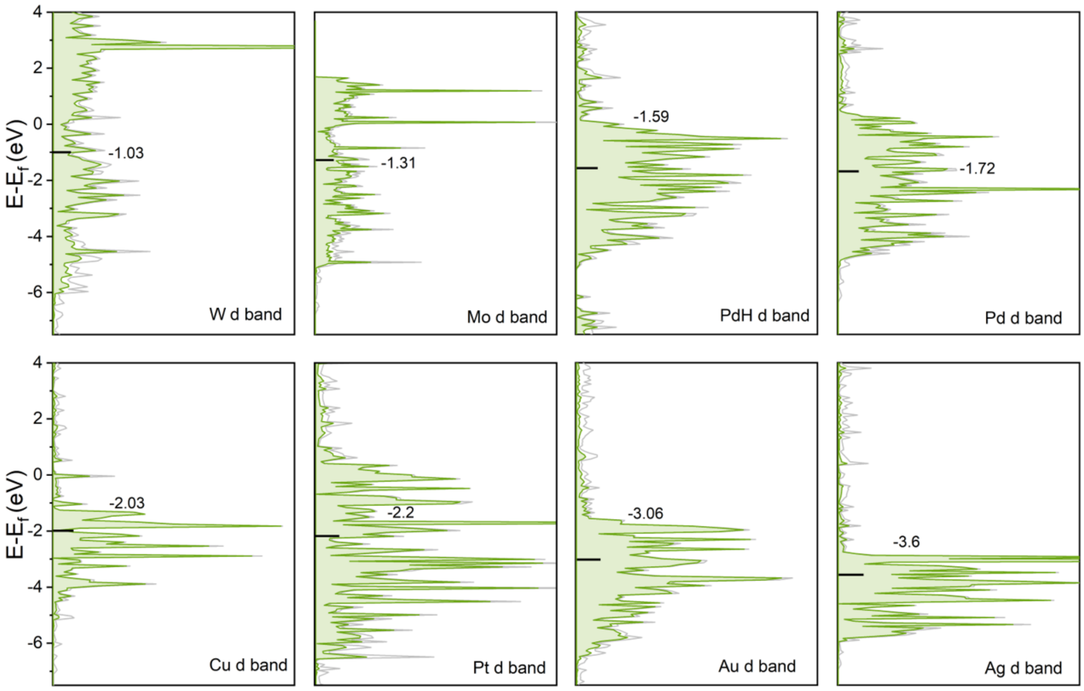

Supplementary Fig. 42. Density of states projected onto the *d* states of W (110), Mo (110), Pd (111), PdH_x (111), Cu (111), Pt (111), Au (111) and Ag (111).

Supplementary Table 1. Price of feedstocks and products.

Product	Price($/Ton)	source
	**Feedstocks**	
Acetonitrile (AN)	1500	b
Water	0.22	a
H_2SO_4	45	a
Ethylamine (EA)	10000	b
H_2	1900	a

a: Zhou, H., Ren, Y., Li, Z. et al. Electrocatalytic upcycling of polyethylene terephthalate to commodity chemicals and H_2 fuel. *Nat. Commun.* **12**, 4679 (2021).

b: Taken from online trade market. (https://jskolod.en.made-in-china.com/product/aFlJndtVYuch/China-Potassium-Diformate-98-.html).

Supplementary Table 2. Swelling rate of proton exchange membrane at current density of 200 mA cm^{-2}.

Sample	Reaction Time	Swelling rate in H_2O	Swelling rate in 0.5 M H_2SO_4+8wt% MeCN
Membrane without catalyst	0h	10.2%	18.4%
Membrane with anode catalyst	0h	6.3%	11.6%
Membrane with anode catalyst	5h	5%	12.5%
Membrane with anode catalyst	20h	5.3%	11.8%

Supplementary Table 3. Number of available exchange sites to proton per gram of Nafion for different reaction time at current density of 200 mA cm^{-2}.

Sample	Reaction Time	The proportion of sulfur (%)	Exchange sites per gram (mmol SO_3^-/g)
Membrane with anode catalyst	0h	4.399%	1.37
Membrane with anode catalyst	5h	3.816%	1.19
Membrane with anode catalyst	10h	4.281%	1.34
Membrane with anode catalyst	20h	4.472%	1.4

Supplementary Table 4. EXAFS fitting parameters at the Pd K-edge for Pd/C catalysts at different potentials vs. RHE in 0.5 M H_2SO_4 solution. (S_0^2=0.816).

Potential	Shell	N^a	R(Å)^b	σ^2(Å^2)^c	ΔE_0 (eV)^d	R factor
+0.2 V	Pd-Pd	8.17	2.73	0.00829	2.665	0.006
+0.1 V	Pd-Pd	7.74	2.74	0.00805	2.861	0.008
0 V	Pd-Pd	8.19	2.80	0.00948	3.120	0.010
-0.2 V	Pd-Pd	8.52	2.81	0.01003	2.863	0.010
-0.5 V	Pd-Pd	8.34	2.80	0.00930	3.033	0.005

$^a N$: coordination numbers; $^b R$: bond distance; $^c\sigma^2$: Debye-Waller factors; $^d\Delta E_0$: the inner potential correction. R factor: goodness of fit. S_0^2 was set to 0.816, according to the experimental EXAFS fit of Pd foil reference by fixing coordination numbers as the known crystallographic value.

Supplementary Table 5. EXAFS fitting parameters at the Pd K-edge for Pd/C catalysts at different potentials vs. RHE in 8 wt% MeCN + 0.5 M H_2SO_4 solution. (S_0^2=0.816).

Potential	Shell	N^a	R(Å)^b	σ^2(Å^2)^c	ΔE_0 (eV)^d	R factor
+0.2 V	Pd-Pd	8.47	2.73	0.00883	2.658	0.003
+0.1 V	Pd-Pd	8.11	2.74	0.00844	2.784	0.003
0 V	Pd-Pd	8.02	2.74	0.00791	2.671	0.002
-0.2 V	Pd-Pd	8.43	2.78	0.00952	3.736	0.011
-0.5 V	Pd-Pd	8.37	2.79	0.00954	3.298	0.009

^aN: coordination numbers; ^bR: bond distance; $^c\sigma^2$: Debye-Waller factors; $^d\Delta E_0$: the inner potential correction. R factor: goodness of fit. S_0^2 was set to 0.816, according to the experimental EXAFS fit of Pd foil reference by fixing coordination numbers as the known crystallographic value.

Supplementary Table 6. DFT calculated binding energies (BE) of adsorbates without H coverage on Pd(111), PdH_x(111), Cu(111), Pt(111), Au(111), Ag(111), W(110) and Mo(110) surfaces.

Intermediates	BE/eV							
	Pd (111)	PdH_x (111)	Cu (111)	Pt (111)	Au (111)	Ag (111)	W (110)	Mo (110)
$*CH_3CN$	-0.580	-0.497	-0.300	-0.623	-0.024	-0.049	-0.983	-0.907
$*CH_3CHN$	-2.392	-2.276	-1.90	-2.220	-1.184	-1.147	-4.0183	-3.931
$*CH_3CNH$	-2.595	-2.546	-1.70	-2.886	-1.425	-1.073	-3.833	-3.670
$*CH_3CH_2N$	-3.698	-3.528	-2.853	-3.332	-1.699	-1.562	-5.249	-5.120
$*CH_3CNH_2$	-3.903	-3.844	-3.1442	-4.30	-2.292	-1.678	-4.340	-4.289
$*CH_3CHNH$	-0.976	-0.90	-0.636	-1.17	-0.461	-0.357	-1.259	-1.166
$*CH_3CH_2NH$	-2.233	-2.101	-1.861	-2.24	-1.333	-1.279	-3.342	-3.279
$*CH_3CHNH_2$	-2.084	-2.048	-0.995	-2.307	-0.991	-0.526	-2.52	-2.432
$*CH_3CH_2NH_2$	-0.79	-0.72	-0.459	-0.971	-0.368	-0.2475	-1.011	-0.920
*H	-0.67	-0.615	-0.226	-0.508	0.176	0.209	-0.751	-0.753

Supplementary References

1. Lum Y. *et al.* Tuning OH binding energy enables selective electrochemical oxidation of ethylene to ethylene glycol. *Nat. Catal.* **3**, 14-22 (2020).
